# MSF-DiffDet: A U-Shaped Spatial-Frequency Interactive Network for Diffusion-Generated Handwriting Deepfake Detection

**DOI:** 10.3390/s26216682

**Published:** 2026-10-22

**Authors:** Dawei Liang, Meng Wu, Chen Liu

**Affiliations:** 1 College of Computer, Nanjing University of Posts and Telecommunications, Nanjing 210023, China; liangdawei@jspi.edu.cn; 2 Department of Computer Information and Cyber Security, Jiangsu Police Institute, Nanjing 210031, China; liuchen1@jspi.edu.cn

**Keywords:** handwriting forgery detection, diffusion model forensics, spatial-frequency fusion, learnable frequency decomposition, cross-domain generalization

## Abstract

Recent diffusion models can synthesize handwriting images with sufficient visual fidelity to challenge both human examiners and automated forensic systems, thereby undermining the reliability of document authentication. Existing spatial-domain detectors may fail to capture subtle generation artifacts embedded in complex stroke structures. To address this limitation, we propose MSF-DiffDet, a multi-scale spatial-frequency framework that jointly learns forensic cues from spatial and frequency representations. The framework employs Learnable Frequency-Aware Decomposition (LFAD) to extract frequency-aware features, Local Frequency Statistics (LFS) to characterize local spectral variations, MixBlock to enable hierarchical cross-domain interaction, and an Adaptive Fusion Skip-connection (AF-Skip) decoder to suppress less informative features during response-map generation. MSF-DiffDet is evaluated under intra-domain detection, image-degradation robustness, and zero-shot cross-generator settings across multiple handwriting generators. An additional joint-domain test with Chinese handwriting is included to examine behavior under simultaneous script, generator, and dataset shifts. It outperforms the representative baselines, achieving 93.49% accuracy, 96.30% AUC, and 10.45% EER in the intra-domain evaluation, while retaining 89.16% accuracy under zero-shot cross-generator evaluation on Test Set B. In a strict leave-one-generator-out (LOGO) setting, where the model is trained on DiffBrush and evaluated on WordStylist with writer-disjoint splits, MSF-DiffDet achieves the highest cross-generator AUC (84.74%) among the compared architectures, while the purely spatial and purely frequency-domain variants reach 81.68% and 77.90%, respectively. The gap between in-generator and cross-generator performance indicates that diffusion-generated handwriting detection remains partially generator-specific. A deletion-based faithfulness analysis further shows that high-response regions of the response map are functionally relevant to the image-level prediction. These results demonstrate the effectiveness of spatial-frequency feature interaction for diffusion-generated handwriting detection and for producing forgery-sensitive stroke-region response maps.

## 1. Introduction

Offline handwriting, as a complex behavioral biometric, conveys highly individual-specific physical mechanics and writing habits. In high-security domains such as forensic document examination and financial authentication, the integrity of handwritten evidence serves as a cornerstone for identity verification [1,2]. Traditional handwriting analysis operates under the fundamental assumption that all specimens originate from genuine human writing, possessing biomechanical uniqueness and temporal stability [3].

Before the emergence of generative artificial intelligence, researchers in the fields of both handwriting detection and synthesis assumed that text generated by writing style mimicry could be distinguished from genuine handwriting. Indeed, while some early attempts at writing style mimicry, based on generative adversarial network (GAN) techniques, could approximate an overall writing style, the artificial samples usually suffered from mode collapse and visible artifacts, hence were easy to identify [4,5]. However, recent advancements in synthetic handwriting, based on denoising diffusion probabilistic models (DDPMs) [6,7,8] and their latent-space-variant versions (LDMs) [9], have overhauled this assumption. Recent studies have shown that LDM-based handwriting generators can produce realistic text-level and word-level handwriting samples with coherent strokes and ink texture that are difficult for human viewers to differentiate from genuine samples, as well as for handwriting recognition systems [8,9].

The threat of synthetic handwriting has started to trigger research interest. Carrière et al. [10] showed the feasibility of distinguishing genuine from diffusion-generated handwriting using a standard ResNet18 classifier, with around 90% accuracy. They also showed that the main discriminative cues used by spatial-domain classifiers to perform this task were due to real-world physical artifacts present in the real samples such as ink bleeding, paper texture, etc., and do not properly reflect the intrinsic fingerprints of the generation process, which therefore still leaves the discrimination problem widely open.

More broadly, current AI-generated handwriting detectors face several interconnected limitations. Spatially, RGB-only methods capture macroscopic texture discrepancies but may miss microscopic frequency cues hypothesized to arise from iterative denoising [11,12,13]. Recent independent studies have reported systematic frequency-domain anomalies in diffusion-generated imagery, including mid-band reconstruction errors [14] and violations of the natural $1/f^{\beta }$ power-law decay [15]. This blind spot compromises robustness: JPEG compression, Gaussian blur, and rescaling lose subtle spatial clues and often result in significant performance degradation [16]. From a design point of view, single-stream global-pooling classifiers do not have multi-scale integration required to detect local stroke anomalies and global structural failures [17]. In terms of deployability, current detectors provide only binary labels, and lack spatial evidence for forgery localization, which is a problem for forensic workflows that require interpretability [18,19].

The main contributions of this paper are summarized as follows:

(1) We propose MSF-DiffDet, a U-shaped spatial-frequency interactive network for detecting diffusion-generated handwriting. Unlike prior detectors that treat spatial and frequency cues in separate branches, MSF-DiffDet couples the two representations at multiple scales through a shared encoder-decoder topology, enabling stroke-level and spectral evidence to reinforce each other during feature aggregation.

(2) We design four dedicated components that address different aspects of handwriting forensics. The learnable frequency-aware decomposition (LFAD) separates an input into complementary spectral subbands. The local frequency statistics (LFS) module characterizes their neighborhood behavior. MixBlock introduces hierarchical interaction between the spatial and frequency pathways. AF-Skip provides an adaptive skip-fusion path in the decoder for classification-oriented feature aggregation.

(3) We build a multi-generator evaluation benchmark (WordStylist, DiffBrush, TGC-Diff), where Test Set B isolates generator shift under the same Latin script and IAM family, and Test Set C captures a joint-domain shift involving script, generator, and dataset changes simultaneously. We further conduct a strict leave-one-generator-out (LOGO) experiment (train on DiffBrush, test on WordStylist, writer-disjoint) to directly examine cross-generator generalization. MSF-DiffDet reaches 93.49% accuracy, 96.30% AUC, and 10.45% EER intra-domain, retains 89.16% accuracy on the unseen DiffBrush generator, and achieves the highest cross-generator AUC (84.74%) among the compared architectures in the LOGO setting, while remaining competitive under common image degradations.

(4) We introduce a deletion-based faithfulness analysis to examine whether the forgery-sensitive stroke-region response is coupled to the image-level forgery decision. Removing high-response regions reduces the forged-class score more rapidly than unrestricted random deletion, providing evidence that the highlighted regions carry information relevant to the classification output. The analysis is treated as preliminary faithfulness evidence rather than a claim of precise artifact-level localization, since no pixel-level artifact annotation is available.

This paper is structured as follows: Section 2 surveys recent advances in handwriting deepfake detection research. Section 3 presents a detailed description of the proposed method. Section 4 introduces the datasets and reports the experimental results. Section 4.4 further analyzes the faithfulness of the forgery-sensitive stroke-region response through three complementary control experiments. Finally, Section 5 concludes and outlines future research directions.

## 2. Related Work

This section reviews the literature along three threads that collectively frame the research context of this study: the evolution of handwriting generation techniques, which defines the adversarial capability; mainstream AI-generated image detection methods, which represent the current defensive landscape; and spatial-frequency fusion strategies for image forensics, providing the technical foundation for our approach. Through the analysis of each thread, we identify the specific limitations of existing methods when applied to structurally sparse handwriting images and thereby motivate the proposed multi-scale spatial-frequency interactive detection framework.

### 2.1. Handwriting Generation

Handwritten text generation (HTG) aims at creating offline or online handwritten images that preserve both target writer style and text. The field has developed in three main phases (recurrent networks, adversarial networks, diffusion models) whose visual realism is improved and the stakes for downstream forgery detection are high.

Early neural approaches treated handwriting as temporal stroke sequences. Graves [20] showed that Long Short-Term Memory (LSTM) networks conditioned on character sequences can generate plausible online handwriting trajectories, demonstrating the feasibility of data-driven writing synthesis. However, these recurrent methods are designed for online stroke generation and do not directly produce the raster images typically encountered in document forensics.

The pixel-level image generation was shifted towards the pixel-level image generation with GANs [21]. ScrabbleGAN [22] introduced a recurrence-free semi-supervised framework synthesizing variable length word images by overlapping character-level receptive fields end-to-end. GANwriting [23] introduced writer-level style conditioning with a few-shot mechanism for personalized style imitation from limited reference samples. Davis et al. [24] extended generation from isolated words to full text lines using style and content conditioning. HWT [25] incorporated Transformer-based self-attention into generation to capture long-range structural dependencies to reduce geometric distortions common in long-sequence outputs. JokerGAN [26] explored memory-efficient architectures with text-level conditioning for scaling up deployment.

Although GAN-based methods often suffered from mode collapse and broken stroke connectivity (especially for characters with complex topologies such as Chinese ideographs), they were not always able to provide the typefaces (because adversary optimization is unstable). DDPMs have enabled handwriting generation to be more precise [27]. Luhman and Luhman [28] demonstrated early that diffusion can produce plausible handwriting samples. WordStylist [29] built on Latent Diffusion Models obtained fine-grained conditional disentangling of calligraphic style traits (stroke width, slant, ink intensity) while maintaining text quality. DiffBrush [30] defined the writing process as continuous denoising trajectories of a physical brush model yielding smooth strokes with minimal geometric distortion. TGC-Diff [31] further introduced a template-guided cascade diffusion architecture constraining local character skeletons while maintaining global text line layout. This significantly improved synthesis quality for long sequences.

While these advances have made generative models capable of producing handwriting with considerably higher visual realism, they have also lowered the barrier for malicious applications—forged signatures, altered financial instruments, fabricated historical documents—creating a clear need for high-precision detection technologies that can distinguish genuine from synthesized specimens.

### 2.2. AI-Generated Image Detection

As generative models become more capable, detecting their outputs has become a major area of research in computer vision and digital forensics. Existing detection methods are classified by the signal domain they exploit.

Spatial-domain methods focus on pixel-level inconsistencies or visual artifacts [32,33]. Wang et al. [34] showed that ResNet-based binary classifier trained on ProGAN outputs with blur and JPEG enhancement can generalize to unseen GANs. Ojha et al. [35] used CLIP vision-language representations to build universal detector that transfers to different generators without tuning them. Yu et al. [36] showed that each GAN instances leave distinct fingerprints in outputs, both detection and source attribution.

Compact spatial models originally designed for face forgery (Xception [37] and MesoNet [38]) are also used as effective starting points for detecting synthetic images by local textural differences. These models were developed for semantically dense facial regions and not specifically designed for sparse stroke layouts of handwriting. Generally, spatial domain approaches assume dense image content. When applied to handwriting where foreground strokes are only small traced with large blank backgrounds, they face a “feature dilution” problem where informative signals are overwhelmed by uninformative background regions.

Frequency-domain methods exploit the observation that generative networks introduce systematic biases to upsampling and deconvolution. Frank et al. [11] showed early that GAN-synthesized images have periodic high-frequency artifacts in their Fourier spectra. Durall et al. [39] showed that up-convolution operations in deep generators do not reproduce the correct roll-off of natural images, which is detectable by power spectral density analysis. Dzanic et al. [40] classifies these Fourier spectrum differences as light discriminative feature set, while Corvi et al. [41] also found that iterative denoising schedules leave detectable frequency-band artifacts distinct from GANs. These methods provide generator-agnostic forensic evidence at the distribution level, but typically depend on global spectral statistics and are not sensitive to spatial locality, which is a critical limitation for handwriting where discriminative artifacts concentrate along sparse stroke edges rather than spreading uniformly across images.

Detection methods for the denoising process have been proposed [42]. DIRE [43] (Diffusion Reconstruction Error) passes a query image in the forward-reverse pipeline of a trained diffusion model and measures the pixel-level reconstruction error. Since diffusion models “memorize” their own generative manifold, synthesized inputs are reconstructed more faithfully than real photographs. We can use the gap as detection signal [44] (DE-FAKE) to detect text-to-image outputs. Although they can detect text-to-image outputs, they rely on global reconstruction statistics or whole-image aggregation, and are not effective on handwriting, where the signal occupies only a small fraction of the image area.

Beyond document and handwriting forensics, deepfake detection is a broad research area spanning multiple modalities and application domains. In the face anti-spoofing (FAS) community, for instance, recent methods aim to distinguish genuine facial presentations from synthetic or replayed attacks. A representative work is mmFAS [45], which addresses the latent feature gap across heterogeneous visual modalities (RGB, depth, and infrared) by jointly employing instance-level contrastive learning and class-level matching for multi-level alignment, followed by a switch-attention fusion module that aggregates complementary information while controlling model complexity. While face-centered methods such as mmFAS operate on rich multimodal facial data with dense semantic cues, our task targets sparsely-inked handwritten text, where the discriminative evidence lies in stroke-level spectral artifacts and local boundary irregularities rather than facial semantics. Nevertheless, both directions share the common goal of detecting AI-generated synthetic content, and the principle of explicit alignment before fusion in mmFAS offers a conceptual parallel to the spatial-frequency interaction design in MSF-DiffDet.

In the specific context of handwriting forensics, Carrière et al. [10] conducted the first study targeting diffusion-generated handwritten text. By fine-tuning a ResNet-18 classifier on binarized genuine/WordStylist image pairs, they achieved 89.57% accuracy and observed that the learned features partially transfer to other generator architectures (e.g., GANwriting). Their analysis suggested that the primary discriminative signal stems from physical scanning and ink-deposition artifacts present in genuine samples rather than from spectral fingerprints of the generative process. However, this reliance on dataset-specific physical properties limits cross-corpus transferability, and the exclusive use of spatial RGB features leaves the frequency-domain signatures inherent to the diffusion denoising trajectory entirely unexploited.

Despite the progress reviewed above, handwritten images present a combination of properties—extremely sparse foreground strokes, complex and variable paper textures, and highly informative high-frequency edge details—that existing general-purpose detectors struggle to accommodate. Spatial methods suffer feature dilution and frequency methods lack spatial grounding; meanwhile, the only handwriting-specific work to date operates in a single domain. Addressing this gap requires a framework that jointly leverages both spatial and spectral representations in a structurally aware manner.

### 2.3. Spatial-Frequency Fusion for Image Forensics

The complementary nature of spatial and frequency representations has motivated a growing body of work on dual-domain fusion for image forensics, moving beyond the single-domain paradigms reviewed above [46].

Qian et al. [47] proposed a two-branch architecture for face forgery detection by removing frequency-aware features with learnable DCT filters in one branch and spatial texture features in the other branch, combining them cross-attentionally. They showed that explicit frequency decomposition can capture artifacts invisible in spatial-only networks. Luo et al. [48] showed that high-frequency components carry generalizable forgery clues across unseen face manipulation methods, highlighting the forensic value of spectral analysis. Liu et al. [49] proposed attention-guided spatial-frequency alignment with learnable frequency masks, yielding strong cross domain generalization for synthetic image detection.

Although most current fusion models combine the two methods only at the last stage of the network (usually late concatenation or element-wise addition), they are not able to preserve fine-grained interactions between local spatial structures and multi-scale frequency band anomalies. No such spatial-frequency methods have been designed or evaluated for handwriting. Handwriting is particularly challenging: informative high-frequency content (stroke edges, ink-paper boundary transitions) is concentrated spatially along narrow foreground areas, while large background contains low-frequency noise from paper texture and scanning artifacts. A naive late-fusion approach would permit background frequency noise to dominate fused representation, obscuring subtle spectral anomalies located at stroke boundaries. This limitation motivates us to design a hierarchical multi-scale bidirectional interaction mechanism where spatial structure constraints and frequency-band anomaly signals refine each other at every encoder-decoder stage, not only at the output.

To summarize, the previous review shows three related gaps: (1) a representational gap where spatial-only methods depend on incidental physical cues while frequency domain generative fingerprints are not used for handwriting; (2) an architectural gap because single stream or late-fusion pipelines weaken spatially sparse forgery along stroke boundaries; (3) an output gap where existing detectors only provide image-level decision without pixel-level forgery localization. These gaps motivate a framework that combines multi-scale bidirectional spatial-frequency interaction and dense prediction. The MSF-DiffDet architecture described in Section 3 addresses all three gaps.

## 3. Proposed Method

This section details the proposed Multi-scale Spatial-Frequency Interactive Network (MSF-DiffDet). Addressing the characteristic that diffusion-generated handwriting is macroscopically highly realistic but possesses inherent statistical anomalies in the microscopic high-frequency domain, this study models forgery detection as a cross-modal consistency reasoning problem.

### 3.1. The Main Architecture

**Figure 1:**
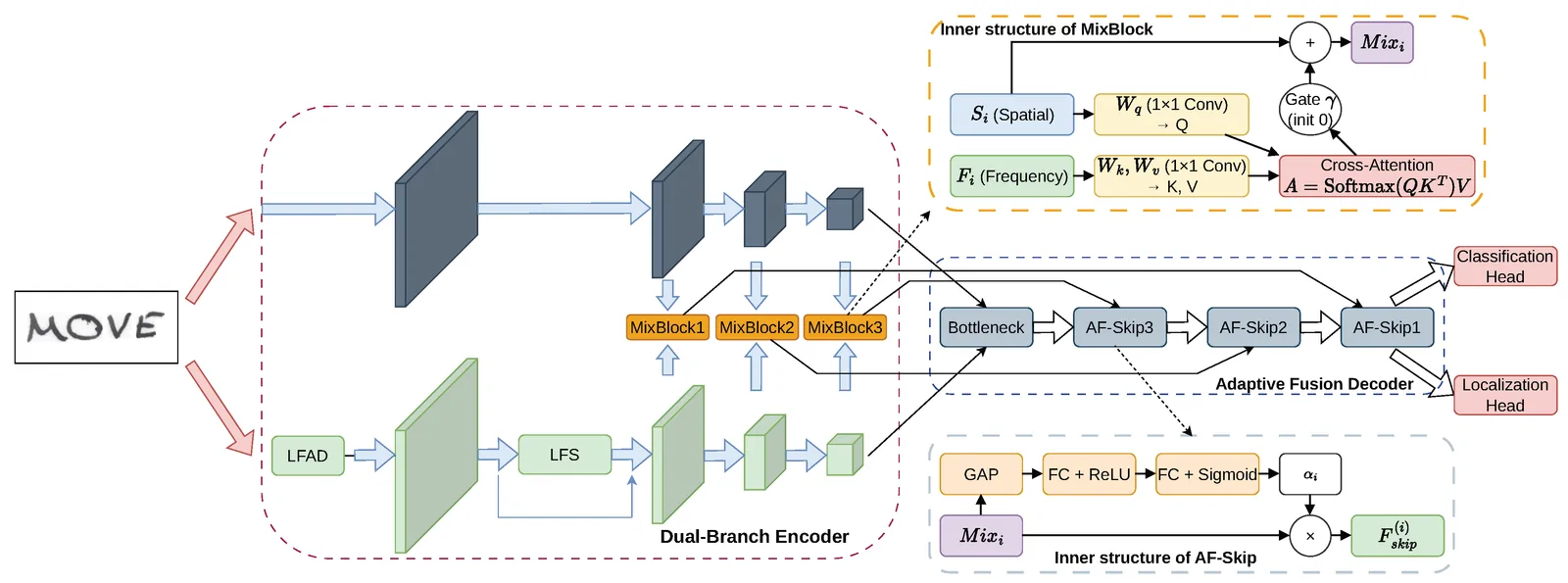
Architecture of MSF-DiffDet. The network contains a dual-branch encoder, multi-scale MixBlock interaction modules, and an adaptive-fusion U-shaped decoder. Insets show the generic inner structures shared by corresponding modules. The upper inset illustrates the cross-attention workflow of MixBlock, and the lower inset shows the channel-attention mechanism inside AF-Skip. LFAD performs FFT-based frequency decomposition and LFS extracts local frequency variance statistics. The model outputs classification prediction and forgery-sensitive stroke-region response map simultaneously.

As illustrated in Figure 1, MSF-DiffDet adopts a hierarchical U-shaped network architecture with decoupled spatial and frequency streams. Unlike traditional single-stream CNN detectors that treat an image as a whole, our processing pipeline is divided into three core stages: (1) Dual-Branch Encoding Stage: Parallel networks extract macroscopic spatial visual structures and explicit microscopic frequency noise; (2) Cross-Domain Hierarchical Interaction Stage: MixBlock modules are embedded at multi-scale encoder levels to actively verify frequency responses using spatial features, achieving geometric alignment of heterogeneous data; (3) Dynamic Adaptive Fusion and Decoding Stage: Utilizing adaptive feature fusion (AF-Skip) and a U-shaped decoding path to suppress background interference, the network synchronously outputs the authenticity probability and a pixel-level forgery-sensitive stroke-region response map.

### 3.2. Dual-Branch Encoder

The corresponding remark is that a CNN has a stronger local induction bias and is more sensitive to high-frequency noise and texture gradients [50]. We use parallel CNN branches to separate the representation of the appearance from the signal.

The spatial stream focuses on the macroscopic features that correspond to human vision—stroke geometry, topology, and ink intensity. Given a single-channel grayscale input image $X\in R^{H\times W\times 1}$, we first apply a 7×7 convolution for initial dimensionality reduction, then we adopt ResNet-50 [51] as the backbone. To support later multi-scale interactions, we extract intermediate feature maps from Stages 2, 3, and 4, denoted as ${\left\{S_{i}\right\}}_{i=1}^{3}$. These feature maps retain spatial location and global layout information, serving as physical anchors for authenticity judgment.

To capture spectral artifacts in diffusion-generated handwriting, the frequency stream employs a Learnable Frequency-Aware Decomposition (LFAD) module inspired by [48]. Given an input tensor $X\in R^{B\times C\times H\times W}$, LFAD applies a two-dimensional real-valued Fast Fourier Transform and uses three independent learnable spectral masks to produce complementary frequency-weighted components:

(1) $$V_{k}=irFFT2\;\left(rFFT2\left(X\right)⊙M_{k},\;s=\left(H,W\right)\right),\;k\in \left\{1,2,3\right\},$$

where rFFT2(·) and irFFT2(·) denote the real-valued 2D FFT and its inverse (implemented via rfft2 and irfft2), respectively, and ⊙ denotes element-wise multiplication in the frequency domain. Each learnable mask $M_{k}\in R^{1\times C\times H\times (W/2+1)}$ is initialized to one and updated during training without explicit normalization, non-negativity, or predefined frequency-band constraints. The three outputs are therefore interpreted as learnable frequency-weighted components rather than strictly predefined low-, mid-, and high-frequency bands. The reconstructed components $V_{1},V_{2},V_{3}$ are concatenated along the channel dimension and processed by the frequency-stream backbone. As visualized in Appendix A.9 (Figure A2), the network adaptively allocates its mask weights to the central spectral regions most discriminative for diffusion-generated handwriting, rather than uniformly amplifying all frequencies.

The reconstructed frequency residual maps explicitly expose high-frequency noise fingerprints that are masked in the spatial grayscale appearance. These are then fed into a parallel ResNet-50 (without sharing weights) to extract hierarchical microscopic frequency features ${\left\{F_{i}\right\}}_{i=1}^{3}$.

To further enhance the detection of texture monotony in diffusion-generated handwriting, a Local Frequency Statistics (LFS) branch is embedded in the shallow frequency encoder (after Layer 1). Diffusion-generated ink strokes often exhibit locally over-smooth textures or periodic micro-patterns that are not salient in single-pixel feature maps but become apparent through neighborhood-level statistics. Given the Layer-1 frequency feature map $F_{1}\in R^{C\times H\times W}$, LFS computes per-channel local variance within a 3×3 sliding window:

(2) $$\mu ={\mathrm{AvgPool}}_{3\times 3}F_{1},\;\sigma^{2}=\mathrm{ReLU}\left({\mathrm{AvgPool}}_{3\times 3}\left(F_{1}^{2}\right)-\mu^{2}\right).$$

The ReLU activates numerically non-negativity. The variance map is processed by 1×1 convolution, batch normalization, and ReLU activation to produce channel-calibrated statistical descriptor, added back to the feature via a residual connection:

(3) $${\hat{F}}_{1}=F_{1}+{\mathrm{Conv}}_{1\times 1}\left(BN\left(\sigma^{2}\right)\right).$$

Encoding local energy fluctuations, the LFS branch finds regions where the frequency stream is abnormally uniform or periodically repetitive responses, characteristic of diffusion generators’ iterative denoising steps.

### 3.3. Cross-Domain Interaction

Traditional dual-stream networks rely on late concatenation of signals from each domain. In order to capture physical differences between spatial and frequency representations, we developed a cross-attention-based MixBlock module that performs deep interactive calibration for multiple encoder levels [52]. The idea is to use spatial features as a guide for retrieving frequency anomalies.

In matrix form, spatial feature serves as Query, and frequency feature serves both Key and Value:

(4) $$Q=W_{q}\cdot S_{i},\;K=W_{k}\cdot F_{i},\;V=W_{v}\cdot F_{i},$$

where $W_{q}$, $W_{k}$, and $W_{v}$ are learnable 1×1 convolution projection matrices. Query and Key projections compress channel dimension by a factor 8 ($d_{q}=d_{k}=C/8$) to balance computation and capacity, while Value projection retains full dimension ($d_{v}=C$). Single-head low-rank design keeps attention matrix compact without losing expressiveness of the retrieved frequency responses. The cross-attention map is computed as:

(5) $$A=\mathrm{Softmax}(Q\cdot K^{T})\cdot V.$$

The spatial Query finds striking stroke-edge structures. The dot product with the frequency Key checks whether high-frequency energy is present or absent at those locations. True strokes usually show natural high-frequency responses at ink boundaries, while diffusion-generated strokes often show smooth or periodic spectral behaviors at the same positions. The cross-attention mechanism assigns higher weights to regions where this spatial-frequency inconsistency is found, extracting discriminating clues from V. A learnable scalar gate initialized to zero modulates attended output before adding it back to the spatial feature via residual connection:

(6) $$Mix_{i}=\gamma \cdot A+S_{i}.$$

This allows the network to start from the spatial representation first in training and gradually add cross domain corrections as training progresses. We apply this interaction at 3 encoder stages (1 to 3), which allows calibration at multiple scales, from local stroke textures and intersecting structures to global layouts. Interacting features highlight spatial-frequency conflicts and isolate suspicious regions that appear plausible but are spectrally anomalous. The module configurations and tensor dimensions are summarized in Table 1.

In terms of computational cost, the low-rank projection (C/8) and progressive downsampling across stages keep the attention overhead manageable. At Mix stage 1, the spatial and frequency features are both of size 256×56×56, and the cross-domain interaction is performed at this 56×56 resolution before the output is downsampled to 512×28×28. The Query and Key projections produce tensors of shape B×32×56×56, while the Value projection retains the full channel dimension and produces B×256×56×56. The resulting attention matrix is therefore computed with a feature dimension of 32 rather than 256, which reduces the matrix-multiplication cost by roughly a factor of eight. At deeper stages, the resolution drops to 28×28 and 14×14, making the attention matrices negligible. Profiling on a single NVIDIA RTX 3090 shows that the three MixBlock modules add about 12% GFLOPs relative to the dual-stream ResNet-50 backbone—a modest overhead given the detection gains reported in the ablation study (Section 4).

### 3.4. Adaptive Fusion Decoder

To reconstruct the dispersed cues obtained from cross-domain interactions into final authenticity predictions and weakly supervised response-map generation, this section constructs a dynamic U-shaped decoder equipped with adaptive fusion skip connections and a global feature bottleneck. At the deepest network layer, the highest-level spatial semantic features and the global frequency spectra converge into the global feature bottleneck. To preserve the complementarity of the dual streams, this bottleneck concatenates the channels and employs a 1×1 convolution to compress the dimensions, fusing spatial and frequency features into a single high-dimensional semantic tensor that provides the fundamental context prior:

(7) $$F_{bottleneck}={\mathrm{Conv}}_{1\times 1}\left(\mathrm{Concat}\left(S_{3},F_{3}\right)\right).$$

During the bottom-up resolution recovery process, skip connections transfer detailed information. Because the mandatory MixBlock interactions capture spatial-frequency conflicts but inevitably introduce some computational noise, an Adaptive Feature Fusion (AF-Skip) module is introduced. Utilizing a channel attention mechanism [53], it dynamically suppresses irrelevant background channels (e.g., paper textures) based on feature response intensities, enhancing the weights of forgery-sensitive regions:

(8) $$\alpha_{i}=\sigma \left(W_{2}\cdot \delta \left(W_{1}\cdot \mathrm{GAP}\left(Mix_{i}\right)\right)\right),$$

(9) $$F_{skip}^{\left(i\right)}=\alpha_{i}⊙Mix_{i},$$

where σ(·) represents the Sigmoid function, ⊙ denotes channel-wise multiplication, and GAP stands for global average pooling. Subsequently, the feature maps undergo bilinear interpolation upsampling, concatenation with AF-Skip outputs, and convolutional decoding. This process acts like reconstructing a puzzle: deep semantics locate the macroscopic contour of suspicious regions, while shallow AF-Skip textures delineate stroke boundaries.

After three decoding and fusion layers, a high-resolution feature tensor integrating spatial structures, frequency-domain information, and cross-stream interactions is obtained. The fused tensor is fed in parallel into two task-specific heads. The classification head applies global average pooling and fully connected layers to produce two-class logits $z_{i}^{\mathrm{cls}}\in R^{2}$. For a mini-batch of *N* samples, the classification loss is the standard cross-entropy loss:

(10) $$L_{\mathrm{cls}}=-\frac{1}{N}\sum_{i=1}^{N}\sum_{c=1}^{2}Y_{i,c}\log\;\left(\mathrm{softmax}\;{\left(z_{i}^{\mathrm{cls}}\right)}_{c}\right),$$

where $Y_{i,c}$ denotes the one-hot image-level label of sample *i*.

The localization head applies a 1×1 convolution followed by bilinear upsampling to produce a single-channel stroke-region response logit map $z_{i}^{\mathrm{loc}}\in R^{H\times W}$. Its corresponding response map is

(11) $${\hat{M}}_{i}=\sigma \;\left(z_{i}^{\mathrm{loc}}\right),$$

where σ(·) is the sigmoid function. Because pixel-level annotations of the manipulated regions are unavailable, the localization branch is trained using an automatically generated, class-conditioned weak stroke-region target. Let $y_{i}\in \left\{0,1\right\}$ denote the image-level label, where $y_{i}=1$ indicates a forged sample. After image resizing and before input normalization, the grayscale image $I_{i}$ on the original 0–255 intensity scale is converted into the target mask

(12) $$M_{i,h,w}^{\left(200\right)}=\left\{\begin{array}{cc} 1, & y_{i}=1\;\mathrm{and}\;I_{i,h,w}<200,\\ 0, & \mathrm{otherwise}. \end{array}\right.$$

Thus, the target is set to zero over the entire image for genuine samples. For forged samples, pixels darker than the fixed grayscale threshold of 200 are assigned to the positive stroke region. The resulting mask is a class-conditioned weak stroke-region supervision target rather than a manually annotated manipulation-region ground truth.

It is important to note that although this target is constructed from the image-level label, the response map objective differs fundamentally from the classification objective. The classifier produces a single global score and has no incentive to localize where the forgery evidence resides. The response map, by contrast, is supervised at every spatial location. Even when two forged samples share the same image label, their response maps can differ substantially because the forgery evidence is spatially distributed differently (see Figure A3). This forces the localization branch to learn region-level spectral traces rather than memorize a global label-conditioned pattern. The two objectives are therefore complementary: the classification loss provides the primary supervisory signal, while the localization loss acts as a spatial regularizer that encourages the shared encoder to preserve fine-grained forensic cues. As demonstrated in Appendix A.10, removing the localization loss (λ=0) causes a 13.75 percentage point drop in the cross-generator LOGO setting, revealing that spatial supervision is particularly valuable for generalization.

The localization loss is the mean pixel-wise binary cross-entropy with logits:

(13) $$L_{\mathrm{loc}}=\frac{1}{NHW}\sum_{i=1}^{N}\sum_{h=1}^{H}\sum_{w=1}^{W}\;\left[\max\;\left(z_{i,h,w}^{\mathrm{loc}},0\right)-M_{i,h,w}^{\left(200\right)}z_{i,h,w}^{\mathrm{loc}}+\log\;\left(1+\exp\;\left(-\left|z_{i,h,w}^{\mathrm{loc}}\right|\right)\right)\right].$$

Equation (13) is the numerically stable binary-cross-entropy-with-logits formulation used in implementation. The model is trained using the joint objective

(14) $$\Theta^{*}=\arg\min_{\Theta }E_{B∼D}\;\left[L_{\mathrm{cls}}\left(B\right)+\lambda L_{\mathrm{loc}}\left(B\right)\right],$$

where B denotes a mini-batch sampled from the training set and λ controls the contribution of the localization term. Both losses contribute gradients to their task-specific branches and to the shared feature-extraction layers involved in their computation. Accordingly, ${\hat{M}}_{i}$ is interpreted as a weakly supervised forgery-sensitive stroke-region response associated with the forged-image decision, rather than a pixel-level ground-truth segmentation of the manipulated regions.

## 4. Experiments

This section evaluates in-domain detection, robustness to image degradation, cross-domain generalization, and module contributions. Attribution and failure-case analyses are also provided.

### 4.1. Datasets and Preprocessing

#### 4.1.1. Datasets and Test Sets

Since no established benchmark is available for diffusion-generated handwriting detection, we construct Dataset A from the IAM handwriting database [54]. Each genuine IAM word image is paired with a forged image generated by WordStylist [29] using the corresponding text transcription and handwriting style condition. Dataset A contains 30,512 images, comprising 15,256 genuine samples and 15,256 generated samples.

The IAM database contains 657 writers in total. To avoid writer-specific leakage, the writer identity is extracted from the IAM word IDs. For Dataset A, we use 600 writers. The 600 Dataset-A writers are partitioned into training, validation, and Test-A groups at a ratio of 8:1:1 using a fixed random seed of 42, i.e., 480 training writers, 60 validation writers, and 60 Test-A writers. All samples associated with the same writer are assigned to only one subset. Each WordStylist-generated image follows the split assignment of its corresponding IAM source sample. Consequently, the three subsets are writer-disjoint and retain a 1:1 ratio between genuine and generated images. The resulting split contains 24,408 training images (480 writers), 3052 validation images (60 writers), and 3052 Test-A images (60 writers). Because the partition is performed at the writer level, the exact image proportions may differ slightly from 8:1:1. Unless otherwise stated, Test Set A denotes the held-out writer-disjoint test split of Dataset A.

Two additional test sets are used to evaluate distribution shifts. The remaining 57 IAM writers are exclusively reserved for Test Set B. Test Set B contains 2000 genuine IAM word images from these 57 writers and 2000 images generated by DiffBrush [30] under IAM-based style conditions. The 57 Test-B writers are strictly disjoint from all Dataset-A training, validation, and Test-A writers, and the writer-level intersection is zero. Test Set B is used to evaluate cross-generator transfer from WordStylist to DiffBrush. Test Set C contains 2000 genuine Chinese handwriting images from HWDB2.2 [55] and 2000 Chinese handwriting images generated by TGC-Diff [31]. The genuine HWDB2.2 images come from 170 distinct writers. Since HWDB2.2 and IAM are independent databases with completely separate writer pools, there is zero writer overlap between Test Set C and Dataset A. Test Set C introduces a joint domain shift involving the handwriting script, generative model, and genuine-image source, together with potential differences in acquisition and preprocessing characteristics. It therefore varies multiple factors simultaneously and should not be interpreted as an isolated evaluation of script-specific generalization. Table 2 summarizes which factors vary across the evaluation settings.

The three generators are used with their official pre-trained checkpoints without fine-tuning on our data: WordStylist (word-level, trained on IAM), DiffBrush (text-line, trained on IAM), and TGC-Diff (text-line, trained on a Chinese text-line dataset). Because DiffBrush and TGC-Diff generate text lines whereas the paired genuine samples are word-level or character-level images, we apply a granularity alignment procedure: DiffBrush-generated text lines are segmented into word-level images and paired with genuine IAM word images; TGC-Diff-generated Chinese text lines are segmented into character-level images and paired with genuine HWDB2.2 character images. Segmentation is applied identically to genuine and forged text lines, and only samples with matched segmentation results on both sides are retained. This symmetric procedure ensures that genuine and forged samples share identical preprocessing within each pair and that segmentation-pipeline asymmetry cannot be the dominant discriminative cue. Full generator configurations, including checkpoint identifiers, samplers, sampling steps, guidance scales, generation resolutions, and random seeds, are reported in Appendix A.4 (Table A3).

To further ensure data quality, we applied perceptual hash (phash) duplicate checking to all test partitions. Image pairs with a phash Hamming distance of less than 5 are treated as near-duplicates and removed, preserving the 1:1 genuine-forged ratio. After duplicate checking, Test Set B retains 1983 genuine-forged pairs (3966 images), and Test Set C retains 1999 genuine-forged pairs (3998 images). The small number of removed pairs indicates that the original test sets contain very few near-duplicates.

#### 4.1.2. Leave-One-Generator-Out (LOGO) Split

To directly examine cross-generator generalization, we construct a strict leave-one-generator-out (LOGO) benchmark in addition to Test Sets A, B, and C. The training and validation sets are drawn from DiffBrush-generated handwriting, whereas the test set is drawn from WordStylist-generated handwriting. The two generators are never seen together during training. The writer sets of the three splits are fully disjoint, so the benchmark enforces both generator-disjoint and writer-disjoint conditions. Specifically, the LOGO training set contains 20 writers and 8564 word pairs, the validation set contains 8 writers and 3826 word pairs, and the test set contains 41 writers and 9000 word pairs. Writers with fewer than 100 word pairs on the DiffBrush side and fewer than 30 word pairs on the WordStylist side are excluded to avoid unreliable splits. The LOGO split is used only for the cross-generator analysis in Section 4.6 and Appendix A.8; it does not affect the intra-domain results in Table 3 or the cross-generator and joint-domain results reported in the corresponding cross-generator table.

**Figure 2:**
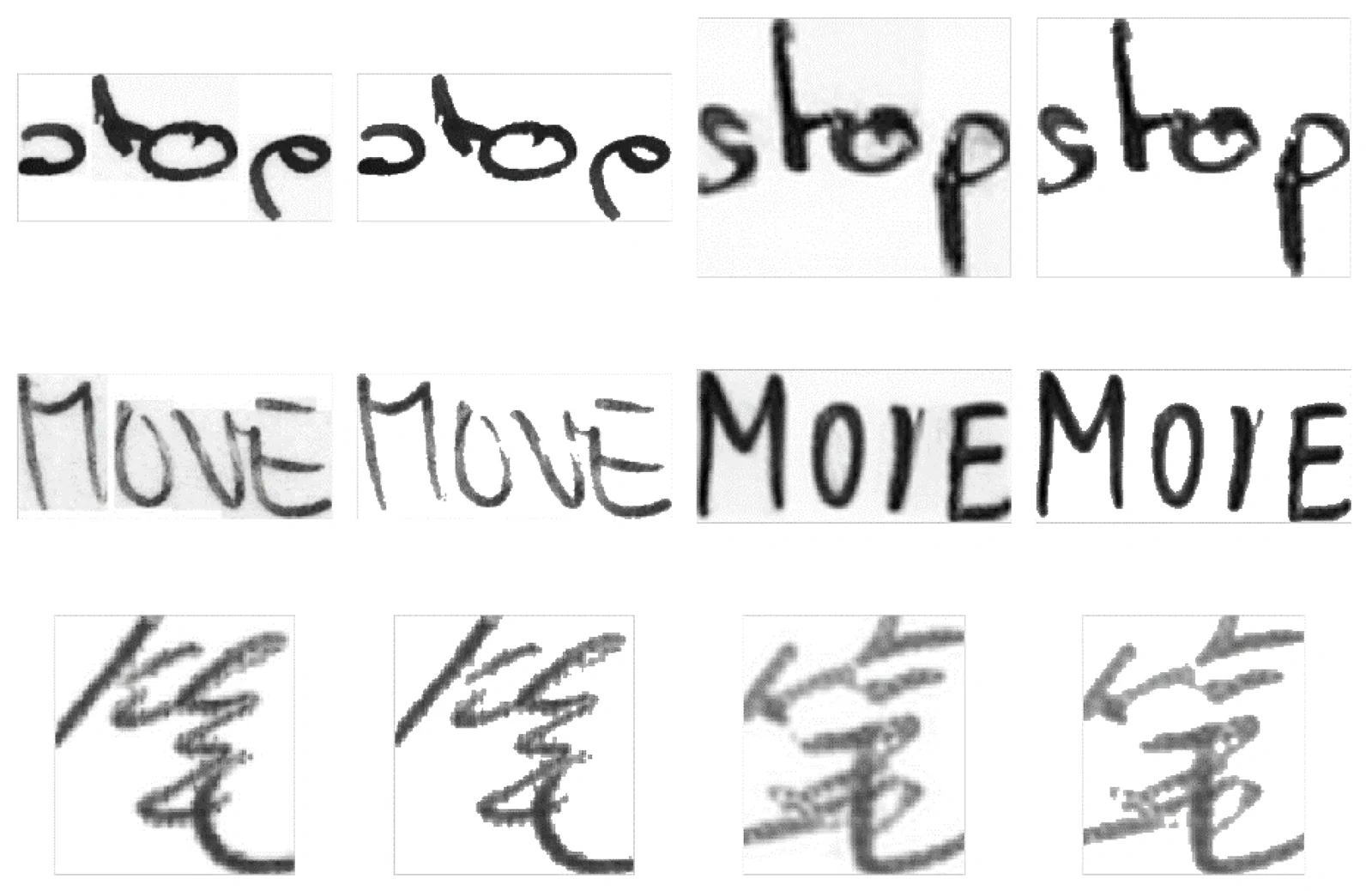
Representative genuine and generated handwriting samples from Dataset A, Test Set B, and Test Set C. The rows correspond to WordStylist, DiffBrush, and TGC-Diff, respectively. For each dataset, the original grayscale images and the images after fixed-threshold stroke-preserving background normalization are shown. Background pixels are replaced with white, while the original grayscale intensities of the detected stroke pixels are retained.

For auxiliary weak localization supervision, each training image (in both Dataset A and the LOGO split) is converted to grayscale on the original 0–255 intensity scale. After resizing and before input normalization, a fixed threshold of 200 is applied. For forged samples, pixels with grayscale intensities below 200 are assigned to the positive stroke region, whereas the remaining pixels are assigned to the background. For genuine samples, the target mask is set to zero over the entire image. The resulting class-conditioned binary mask $M^{\left(200\right)}$ serves as a weak stroke-region supervision target for the localization branch. This fixed-threshold procedure is used consistently throughout training. Otsu thresholding is not used to generate the training targets and is not part of the standard model inference pipeline. Figure 2 illustrates sample instances from each dataset.

### 4.2. Implementation Details

All models are implemented in PyTorch 2.5.1 on a workstation equipped with an NVIDIA RTX 3090 GPU. Input images are resized to 224×224 and converted to single-channel grayscale. All images undergo a single-sided binarization step in which the background is replaced with pure white while the original grayscale intensities of the stroke pixels are retained. This preprocessing is applied identically to genuine and forged images. Pixel values are then scaled to the [0,1] range and standardized with mean 0.5 and standard deviation 0.5 before being fed to the network. For the diffusion-based generators, forged images are synthesized at the native resolution of each generator and processed through the identical preprocessing pipeline, so that all compared images share the same input statistics.

Training uses the AdamW optimizer with a weight decay of 0.0001, a batch size of 32, and an initial learning rate of 0.0001, which is decayed via a cosine annealing scheduler over 50 epochs. The balance coefficient λ in Equation (14) is set to 5.0 to balance the contributions of the classification and localization terms during joint optimization. This coefficient scales the localization gradient but does not block its back-propagation. Both losses contribute gradients to their task-specific branches and to the shared feature-extraction layers involved in their computation.

To ensure a fair comparison, all models (including MSF-DiffDet and all baselines) were trained under identical configurations: the same writer-disjoint data splits, input resolution (224×224), optimizer (AdamW), cosine learning rate schedule, batch size, and total training epochs. The training-time data augmentation pipeline is also strictly identical across all models, consisting only of slight random rotation (within $2^{\circ }$) and minor brightness/contrast jitter. No random flipping or cropping is applied, to avoid distorting the topological structure of the handwriting. Crucially, no blur-based augmentation is used during the training of any model. The Gaussian blur results reported in Section 4.5 are obtained purely as post-hoc test-time perturbations applied to the test images. Hyperparameters for all methods were selected from a shared predefined search space based on validation-set performance, without additional tuning iterations or customized augmentation for any specific model.

Model performance is evaluated based on Accuracy, Precision, Recall, F1-Score, AUC, and Equal Error Rate (EER). The EER is the operating point at which the false acceptance rate equals the false rejection rate. All threshold-dependent metrics (FPR, FNR, accuracy, balanced accuracy) are computed using a single fixed threshold $\tau_{val}$ selected on the validation set, which is then applied unchanged to all test sets. AUC and EER are threshold-independent metrics derived from the full ROC curve. Note that at the fixed threshold $\tau_{val}$, FPR and FNR are generally not equal, so their average does not equal the threshold-independent EER. In the following tables, the downward arrow (↓) indicates that lower values denote better performance.

### 4.3. Comparison with Baselines

To verify the effectiveness of MSF-DiffDet, we compare it against a set of representative baselines covering spatial-only, frequency-only, dual-branch fusion, synthetic-image-artifact and foundation-model detectors: (a) ResNet-18 [51], a lightweight spatial-domain convolutional baseline operating only on image-domain features; (b) ResNet-50 (Spatial-Only), a capacity-matched spatial baseline that isolates the effect of deeper backbone capacity; (c) Frequency-Only, a single-branch baseline that uses only the frequency stream of our encoder to evaluate the discriminative power of frequency-domain cues alone; (d) Dual-LateFusion, a dual-branch baseline with the same encoder backbones as MSF-DiffDet but without hierarchical MixBlock interaction, where spatial and frequency features are combined only through late concatenation; (e) Fixed-FFT Dual-Branch, a dual-branch baseline that replaces the learnable LFAD module with a conventional fixed radial bandpass decomposition, in which the input spectrum is partitioned into three non-overlapping frequency bands (low, mid, high) with hard-coded boundaries, while all other network components are kept identical to MSF-DiffDet. This baseline is included to compare learnable frequency decomposition against conventional hand-crafted frequency decomposition; (f) F^3^-Net [47], a representative published spatial-frequency fusion forgery-detection method originally designed for face manipulation. Since the official implementation was not released, we reproduced it faithfully from the original paper description and adapted the input layer to single-channel handwriting images while keeping its core frequency-aware decomposition and attention modules unchanged; (g) CNNDetection [34], a generative image detector that exploits common systematic artifacts in CNN-generated images; and (h) UnivFD [35], a CLIP-based universal detector that leverages large-scale pre-trained visual-language representations. For our reproduced UnivFD baseline, the CLIP ViT-L/14 backbone was kept frozen, and only the downstream classification head was optimized on our handwriting dataset.

These baselines cover a conventional spatial CNN, a capacity-matched deeper spatial network, an isolated frequency stream, a naive late-fusion dual-branch model, a fixed-frequency dual-branch model, an existing spatial-frequency forensic approach, a synthetic-image-artifact detector, and a foundation-model-based universal detector, enabling a comprehensive assessment of where MSF-DiffDet’s gains originate. All models are retrained on the same training split, share the same input preprocessing, and are evaluated under identical conditions. Each baseline follows its literature-recommended or official training protocol. When the official setting was not directly transferable to our single-channel grayscale handwriting inputs, the key hyper-parameters, notably the initial learning rate, were kept at their literature-recommended values. Training stochasticity is accounted for by averaging every result over three independent random seeds, with standard deviations reported directly in Table 3.

Recent diffusion-aware detectors reconstruct an image via forward-reverse diffusion sampling and measure the reconstruction error, including DIRE [43], De-Fake [44], and FIRE [14]. These methods are designed for dense natural or text-to-image content and require a dataset-specific pre-trained diffusion model operating at the full image resolution. Adapting them to our sparse handwriting setting would require training a dedicated IAM-conditioned diffusion model, executing hundreds of forward-reverse sampling steps per test image, and re-tuning the reconstruction threshold. This is a substantial and largely orthogonal engineering effort. Moreover, for stroke-sparse images, the reconstruction signal occupies only a small fraction of the image, which is precisely the regime where whole-image reconstruction statistics are least effective. We therefore do not include these reconstruction-based detectors in the quantitative benchmark, and we instead retain CNNDetection [34] as a nearest-line synthetic-image-artifact control under the identical protocol. A direct quantitative comparison with reconstruction-based diffusion detectors on a handwriting-adapted diffusion model is an important direction that we explicitly defer to future work.

Table 3 indicates that MSF-DiffDet achieves the strongest overall in-domain performance, particularly in AUC and EER. Several observations can be drawn. First, ResNet-50 improves accuracy from 89.85% to 92.76% over ResNet-18, confirming that backbone capacity contributes to performance but does not fully account for the final result. Second, the Frequency-Only baseline reaches 91.69% Acc, showing that frequency-domain cues are informative but insufficient on their own. Third, Dual-LateFusion, which adopts the same dual encoders as MSF-DiffDet but replaces multi-scale MixBlock interaction with simple late concatenation, only reaches 92.64% Acc and remains 0.85 percentage points below our full model. This demonstrates that the hierarchical cross-domain interaction inside MixBlock, rather than dual-branch stacking itself, is the main source of additional gain. Fourth, the Fixed-FFT Dual-Branch baseline, which replaces the learnable LFAD module with a fixed radial bandpass decomposition, achieves 91.35% accuracy and 93.96% AUC. This is approximately 2.1 percentage points below the full MSF-DiffDet model (93.49% Acc, 96.30% AUC) and 2.3 points below on the threshold-independent AUC metric. Detailed complementarity and significance analyses of the learnable frequency decomposition are provided in Appendix A.6.

To assess whether the differences between MSF-DiffDet and each strong baseline are statistically significant rather than attributable to training noise, we performed paired per-image tests on the full Test Set A. AUC differences are evaluated using image-level bootstrap confidence intervals (2000 resamples), and fixed-threshold classification differences are evaluated using the McNemar exact test. Results are reported in Table A4 of Appendix A.5. MSF-DiffDet exhibits statistically significant AUC improvements over all compared baselines, with the 95% bootstrap confidence intervals entirely above zero in every case. The fixed-threshold accuracy improvement is highly significant over ResNet-18, Frequency-Only, $F^{3}$-Net, and CNNDetection (McNemar $p<10^{-4}$ in all cases). For UnivFD, the improvement is also significant. Since the accuracy comparison uses the McNemar exact test, the result is not limited by the bootstrap resampling resolution; we report the McNemar exact test result as $p<5\times 10^{-4}$. Over the two strongest baselines, ResNet-50 (Spatial-Only) and Dual-LateFusion, the accuracy gap is smaller (+0.73 and +0.85 percentage points) and does not reach conventional significance (p=0.074 and p=0.088). We report these *p*-values honestly and do not over-claim. Because fixed-threshold accuracy depends on the chosen operating point, we rely on the threshold-independent AUC and EER as the primary basis for our claims. The AUC improvements over all compared baselines are statistically significant, and the EER differences are consistent with the AUC ranking; EER was not separately tested for statistical significance.

To disentangle performance improvements originating from network capacity versus architectural innovations, we include ResNet-50 (Spatial-Only) and Dual-LateFusion as capacity-aware baselines. ResNet-50 (Spatial-Only) adopts the same backbone depth as the encoders of MSF-DiffDet. Dual-LateFusion shares two ResNet-50 encoders identical to MSF-DiffDet but uses simple element-wise addition for late fusion without MixBlock interaction modules. Full computational overhead metrics (parameters, GFLOPs, inference GPU memory and inference latency) for all baselines are summarized in Appendix A Table A2. Notably, MSF-DiffDet achieves better accuracy than Dual-LateFusion while using slightly fewer parameters (61.83M vs. 62.86M), fewer GFLOPs (22.98 vs. 23.42), lower inference latency (15.13 ms vs. 15.44 ms), and lower peak inference memory (592.8 MB vs. 652.6 MB), confirming that the gains stem from the proposed spatial-frequency interactive design rather than increased model capacity.

### 4.4. Faithfulness of the Forgery-Sensitive Stroke-Region Response

**Figure 3:**
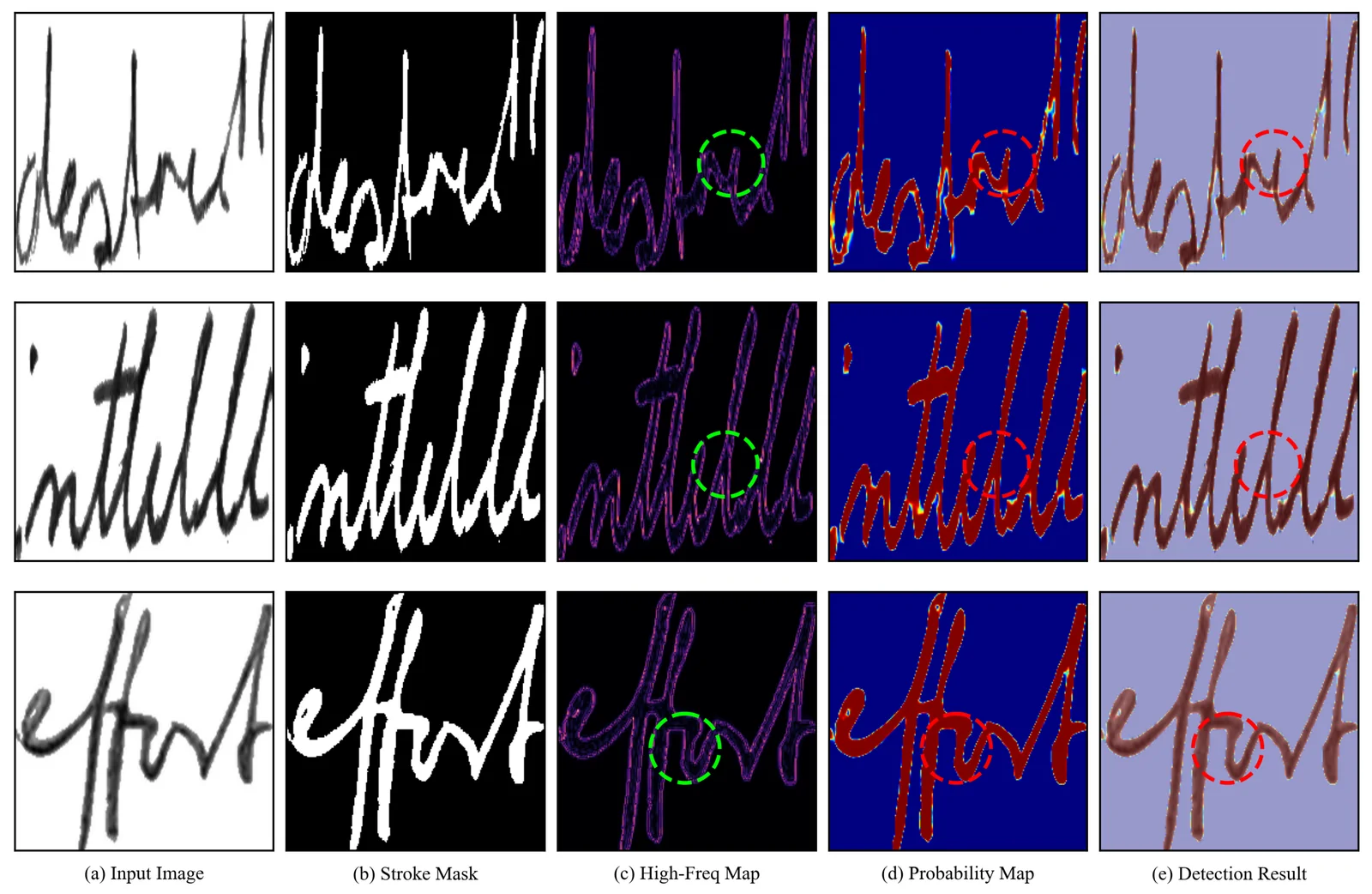
Spatial-frequency interpretability visualization of MSF-DiffDet. From left to right: input image, stroke-region mask generated with the same fixed-threshold procedure as the training target, high-frequency response map, predicted forgery-sensitive stroke-region response map, and final detection result. The displayed binary mask illustrates the stroke-region target-generation procedure and should not be interpreted as manually annotated pixel-level manipulation ground truth. It is distinct from the Otsu-derived domain used only in the foreground-constrained deletion analysis of Figure 4b. The dashed green and red circles highlight representative regions where the model detects local stroke anomalies and predicts forgery-sensitive responses, respectively.

Deep learning models require interpretability for practical forensic applications. Figure 3 qualitatively demonstrates the dual-modal interpretability of MSF-DiffDet. The high-frequency feature maps show strong responses around stroke crossings, high-curvature contours, and local boundary discontinuities. The predicted forgery-sensitive stroke-region response map is concentrated around these local discontinuities and shows limited response to background regions.

The stroke mask displayed in Figure 3 is generated using the same fixed-threshold procedure as the training target $M^{\left(200\right)}$, applied to the input image with a fixed grayscale threshold of 200. It is a visualization of the stroke-region target-generation procedure and should not be interpreted as manually annotated pixel-level manipulation ground truth.

Although Figure 3 shows that the localization branch highlights visually plausible regions, visual plausibility alone does not establish that these regions are associated with the image-level decision. We therefore conducted a perturbation-based deletion evaluation following commonly used faithfulness protocols [56]. Under this protocol, a response map is considered more decision-relevant if removing its highly ranked pixels causes a faster decrease in the forged-class confidence.

Given an input image $x_{i}$, the forgery-sensitive stroke-region response $H_{i}$ and the image-level forged-class probability $p_{i}$ are defined as:

(15) $$H_{i}=\sigma \left(z_{i}^{\mathrm{loc}}(x_{i})\right),\;p_{i}\left(x\right)={\left[\mathrm{soft}\max\left(z_{i}^{\mathrm{cls}}\left(x\right)\right)\right]}_{\mathrm{forged}},$$

where $z_{i}^{\mathrm{loc}}$ and $z_{i}^{\mathrm{cls}}$ denote the localization and classification logits, respectively. We evaluated three pixel-selection strategies. Top-ranked deletion removes pixels in descending order of $H_{i}$, Bottom-ranked deletion removes them in ascending order, and Random deletion uses a uniformly random ordering. The ordering was generated from the original image and remained fixed across deletion ratios within each deletion run. Thus, the deleted pixel sets were nested as the deletion ratio increased.

Two eligible pixel domains were considered. The global domain $\Omega_{i}$ contains all spatial pixels of the image. The foreground-constrained domain $F_{i}^{\mathrm{Otsu}}$ contains only pixels within an Otsu-derived stroke mask. The Otsu-derived foreground mask is used only for the deletion analysis in Section 4.4.2. For strategy *s* and deletion ratio *r*, the first

(16) $$k_{i}\left(r\right)=\mathrm{round}\;\left(r|D_{i}|\right)$$

eligible pixels in the corresponding ordering are selected, where $D_{i}=\Omega_{i}$ for global deletion and $D_{i}=F_{i}^{\mathrm{Otsu}}$ for foreground-constrained deletion. The selected pixels are represented by the binary deletion mask $D_{i}^{s,D}\left(r\right)$. The perturbed input is

(17) $$x_{i}^{s,D,r}=x_{i}⊙\;\left(1-D_{i}^{s,D}\left(r\right)\right)+bD_{i}^{s,D}\left(r\right),$$

where b=1 is the replacement value in the normalized input space. If the preprocessing only scales pixel values from [0,255] to [0,1], b=1 corresponds to the original white-background value of 255.

The experiment used the same 100 randomly selected images from the correctly classified forged samples of Test Set A for both eligible pixel domains. Their mean initial forged-class probability was 0.9999. The evaluated deletion ratios were

(18) R={0,0.05,0.10,0.15,0.20,0.30,0.50}.

For global deletion, a ratio *r* denotes the fraction of all image pixels. For foreground-constrained deletion, it denotes the fraction of pixels within the Otsu-derived stroke mask. Random deletion was independently repeated 20 times for each image and averaged at the sample level. To account for small differences in the initial confidence, each deletion curve was normalized by its undeleted probability:

(19) $$q_{i}^{s,D}\left(r_{j}\right)=\frac{p_{i}\left(x_{i}^{s,D,r_{j}}\right)}{p_{i}\left(x_{i}\right)+\epsilon },$$

where ϵ is a small constant for numerical stability. The image-level normalized deletion AUC was calculated using the trapezoidal rule:

(20) $${\mathrm{AUC}}_{i}^{s,D}=\frac{1}{r_{\max}}\sum_{j=1}^{l}\frac{q_{i}^{s,D}\left(r_{j-1}\right)+q_{i}^{s,D}\left(r_{j}\right)}{2}\left(r_{j}-r_{j-1}\right),\;r_{\max}=0.5.$$

A lower AUC indicates that the selected pixels produce a stronger suppression of the forged-class confidence under the specified perturbation protocol. Because pixel replacement can move an image away from the training distribution, this metric should be interpreted as a relative perturbation-based faithfulness measure rather than a complete causal decomposition of the classifier.

For statistical evaluation, we calculated an AUC for each image and each strategy. We then formed the image-wise paired differences

(21) $$\Delta_{i}^{R-T}={\mathrm{AUC}}_{i}^{\mathrm{Random}}-{\mathrm{AUC}}_{i}^{\mathrm{Top}},\;\Delta_{i}^{B-T}={\mathrm{AUC}}_{i}^{\mathrm{Bottom}}-{\mathrm{AUC}}_{i}^{\mathrm{Top}}.$$

The 95% confidence intervals for the global experiment were estimated using 2000 image-level bootstrap resamples, whereas those for the foreground-constrained experiment were estimated using 10,000 resamples. For each comparison, the within-image differences were calculated before bootstrap resampling, thereby preserving the paired design.

**Figure 4:**
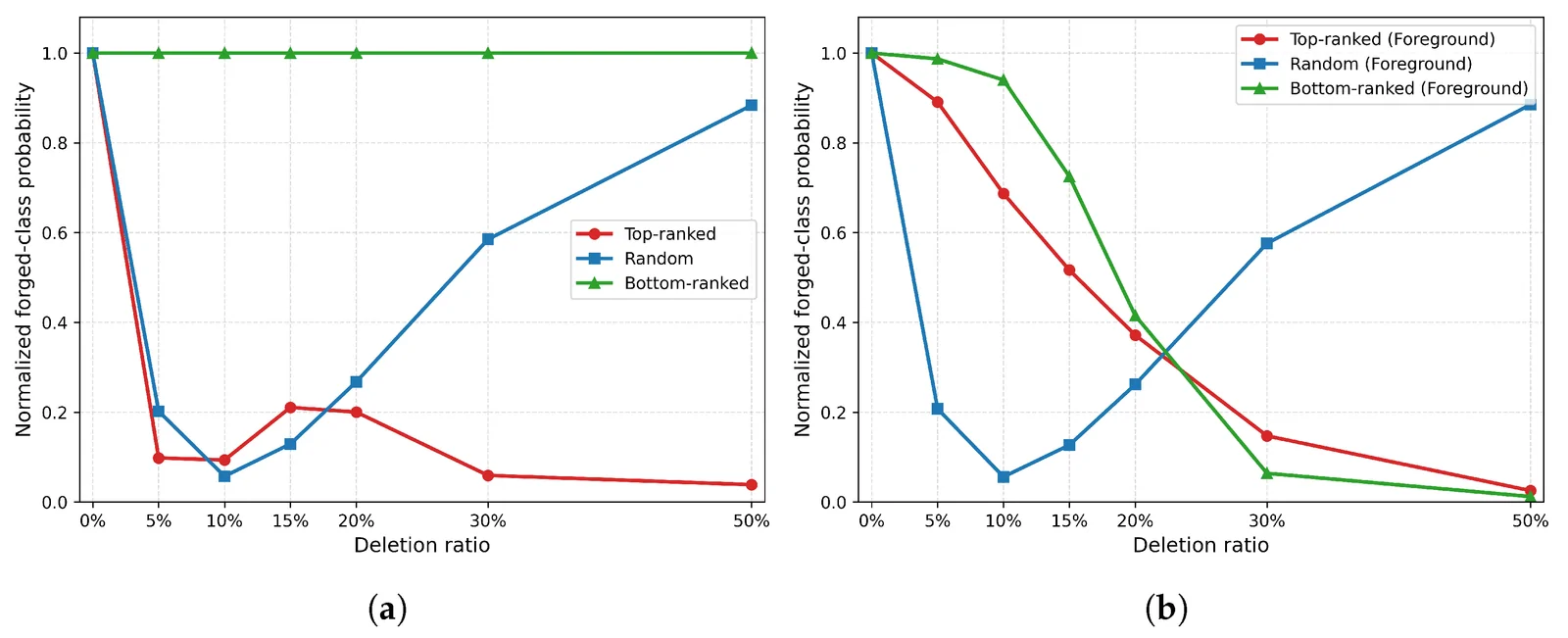
Deletion curves under two eligible pixel domains. (**a**) Global deletion, in which candidate pixels are selected from the entire image. (**b**) Foreground-constrained deletion, in which candidate pixels are restricted to an Otsu-derived stroke domain and then ranked according to the model-predicted forgery-sensitive stroke-region response. The Otsu-derived domain is used only for this evaluation and is not used to train the localization branch or to generate the stroke mask shown in Figure 3. The vertical axis denotes the mean forged-class probability normalized by the undeleted prediction over the same 100 correctly classified forged images. Random deletion was independently repeated 20 times per image and averaged at the sample level. Lower curves indicate a stronger deletion effect under the specified perturbation protocol.

#### 4.4.1. Global Deletion

As shown in Figure 4a, deleting only the top 5% of pixels ranked by the forgery-sensitive stroke-region response reduced the mean normalized forged-class probability from 1.0000 to 0.0983, corresponding to a reduction of approximately 90.17%. In contrast, Bottom-ranked deletion produced almost no change, even when 50% of all image pixels were replaced. This occurs because the lowest localization responses are predominantly located in homogeneous white-background regions, for which replacement with the white-background value produces little or no actual input change.

The deletion curves were not required to be strictly monotonic. MSF-DiffDet is a nonlinear classifier, and extensive pixel replacement can introduce input patterns that differ from the training distribution. We therefore used the AUC over the complete, pre-specified deletion interval as the primary summary statistic.

As reported in Table 4, Top-ranked deletion yielded a normalized AUC of 0.1458, compared with 0.4813 for Random deletion and 1.0000 for Bottom-ranked deletion. The mean paired Random–Top-ranked difference was 0.3355 (95% CI: 0.2920–0.3796), and the mean paired Bottom-ranked–Top-ranked difference was 0.8542 (95% CI: 0.8396–0.8672). Both confidence intervals excluded zero, providing evidence that highly ranked localization pixels produced a stronger deletion effect under the global protocol.

However, the global Bottom-ranked result should be interpreted cautiously. Low-response pixels are predominantly white document-background pixels, and replacing a white pixel with the same white value does not constitute a meaningful perturbation. Moreover, global Random deletion can select many such background pixels. Consequently, the large global separation could partly reflect differences in foreground-background composition rather than only differences in importance within the stroke region.

#### 4.4.2. Foreground-Constrained Deletion

To control for this foreground-background imbalance, we additionally restricted all three strategies to the Otsu-derived foreground stroke mask. Thus, Top-ranked, Random, and Bottom-ranked deletion removed exactly the same number of eligible foreground pixels at each deletion ratio.

As shown in Figure 4b and Table 5, Top-ranked deletion achieved an AUC of 0.3645 (95% CI: 0.3229–0.4078), whereas foreground Random deletion achieved an AUC of 0.4782 (95% CI: 0.4328–0.5244). The image-wise paired Random–Top-ranked difference was 0.1137 (95% CI: 0.0670–0.1614). Because this interval remained above zero, the stroke-region response ranking retained an advantage over random selection even when all deleted pixels were constrained to the foreground strokes. Therefore, the global Top-versus-Random result cannot be attributed solely to Random deletion selecting a larger proportion of white-background pixels.

Top-ranked deletion also had a lower mean AUC than Bottom-ranked deletion, which achieved 0.3991 (95% CI: 0.3713–0.4287). However, the paired Bottom-ranked–Top-ranked difference was 0.0346, with a 95% confidence interval of −0.0035 to 0.0723. Because this interval marginally included zero, the foreground Top-versus-Bottom comparison should be interpreted as a directional trend rather than statistically significant evidence.

The foreground-constrained curves also exhibited pronounced non-monotonic behavior, particularly for Random deletion. Randomly replacing individual pixels inside thin strokes creates isolated white holes, additional local boundaries, and fragmented stroke structures. At low deletion ratios, removal of existing stroke evidence can reduce the forged-class probability. At larger ratios, however, the newly introduced discontinuities can themselves generate high-frequency forensic responses, causing the forged-class probability to rebound. This behavior represents a limitation of independent pixel replacement and illustrates why deletion scores should be interpreted relative to a fixed perturbation protocol rather than as direct causal effects.

Overall, the global and foreground-constrained evaluations provide complementary evidence. The global experiment shows a strong association between high forgery-sensitive stroke-region responses and the image-level forged prediction, whereas the foreground control demonstrates that the Top-versus-Random advantage persists after removing the most direct foreground-background imbalance. Nevertheless, the non-monotonic foreground curves and the inconclusive foreground Top-versus-Bottom comparison indicate that the forgery-sensitive stroke-region response should be interpreted as evidence of classification-response consistency, rather than a complete causal decomposition of the classifier.

We emphasize an intrinsic limitation of our weak supervision setup. Since all stroke pixels of forged samples are assigned positive supervision and genuine samples receive an all-zero target, the localization branch is encouraged to activate on handwriting strokes for forged inputs. Even with the foreground-constrained deletion test, the deletion analysis alone cannot separate stroke-related activations from responses caused by diffusion-specific microscopic artifacts. The above analysis therefore provides correlational rather than causal evidence: high-response stroke regions correlate with the model prediction, but the deletion protocol by itself cannot definitively distinguish diffusion-specific artifacts from general stroke texture patterns.

To further examine the two remaining concerns that the deletion analysis alone cannot resolve, we conducted two additional control experiments, reported in Section 4.4.3 and Section 4.4.4. The first concern is whether the image-level decision depends on background texture rather than stroke content. The second concern is whether the response map merely performs stroke segmentation. The background-only control experiment examines the dependence on stroke-containing regions, and the pure stroke-segmentation baseline examines whether the response map discriminates genuineness beyond stroke segmentation. In the absence of pixel-level ground-truth annotations for diffusion artifacts, the output should nevertheless be strictly interpreted as forgery-sensitive stroke-region response maps instead of precise artifact localization outputs.

#### 4.4.3. Background-Only Control Inference

To further examine whether the image-level decision relies primarily on background texture or on stroke-containing regions, we conducted a background-only control inference experiment on the same 100 correctly-classified forged images used in the deletion analysis. Four input conditions were compared:

- G1 (Original): the full input image;
- G2 (Background-only): all stroke pixels (fixed threshold 200, consistent with the training target $M^{\left(200\right)}$) are erased and replaced with the normalized white value, keeping only background content;
- G3 (Stroke-only): all background pixels are erased and replaced with the normalized white value, keeping only stroke content;
- G4 (Random-mask): an equal number of randomly selected pixels are erased and replaced with the normalized white value, repeated 20 times per image and averaged.

For each condition, we recorded the image-level forged-class probability. The mean ± standard deviation and paired *t*-test results against G1 are summarized in Table 6.

The background-only input (G2) causes a statistically significant drop of 0.1904 in the mean forged-class probability ($p=5.24\times 10^{-18}$), while the stroke-only input (G3) retains 95.66% of the original probability, with only a marginal drop of 0.0433 ($p=1.47\times 10^{-2}$). This indicates that stroke-containing regions carry the dominant forensic signal, while background texture contributes a smaller but non-negligible component.

The random-mask condition (G4) produces a substantially larger drop (0.7470, $p=2.87\times 10^{-37}$), which is lower than the background-only condition. This counter-intuitive result arises because random erasure creates isolated white holes, fragmented stroke boundaries, and additional high-frequency discontinuities that are not present in the training distribution. These perturbation-induced artifacts themselves activate the forensic classifier, and the resulting probabilities therefore do not represent a clean measure of background contribution. We report this result for completeness but caution that G4 should not be interpreted as a causal ablation of stroke content.

Overall, the background-only control experiment provides two pieces of evidence: (i) the image-level forgery decision is primarily driven by stroke-containing regions rather than background texture alone; (ii) the model is not completely invariant to background content, and the residual 0.8096 in G2 suggests that background texture still contributes to the final prediction. We note that this experiment only addresses the foreground-versus-background question. As noted in Section 4.4.2, the weak supervision prevents a full causal separation. This latter question is addressed by the stroke-segmentation baseline experiment in Section 4.4.4.

#### 4.4.4. Stroke-Segmentation Baseline

A natural concern is whether the localization branch merely performs stroke segmentation. To test this, we trained a pure stroke-segmentation baseline (U-Net) using fixed-threshold stroke masks for all images, without using any image-level genuineness label. The baseline is therefore unable to access any information about whether a sample is genuine or forged; it can only learn to segment strokes from image content. We then compared the mean response of the segmentation baseline and the MSF-DiffDet localization branch over stroke pixels of correctly-classified Test Set A samples. The results are summarized in Table 7.

The stroke-segmentation baseline produces almost identical responses on genuine and forged strokes (separation =−0.0003), confirming that a pure stroke segmenter has no capacity to distinguish genuineness. In contrast, the MSF-DiffDet localization branch produces a very low response on genuine strokes (0.0027±0.0332) and a high response on forged strokes (0.9618±0.0372), yielding a separation of +0.9591. The separation is approximately 3200 times larger than that of the segmentation baseline, and the effect-size ratio is $|\mathrm{Cohen}’s\;d_{\mathrm{MSF}}|/|\mathrm{Cohen}’s\;d_{\mathrm{seg}}|\approx 109$.

We note a methodological subtlety in interpreting the Cohen’s *d* of the segmentation baseline. Although the value −0.25 would conventionally fall into the “small” range, it is driven by the extremely narrow dynamic range of the segmentation baseline output: the pooled standard deviation is only about 0.0012, while the raw separation is merely 0.0003. In practical terms, the segmentation baseline produces essentially constant responses on all stroke pixels regardless of genuineness. We therefore recommend interpreting the comparison in terms of raw separation and effect-size ratio rather than Cohen’s *d* alone. By contrast, the MSF-DiffDet localization branch yields a Cohen’s *d* of +27.21, which is well above the conventional “large” threshold of 0.8, confirming its strong capacity to discriminate genuine from forged strokes.

**Figure A1:**
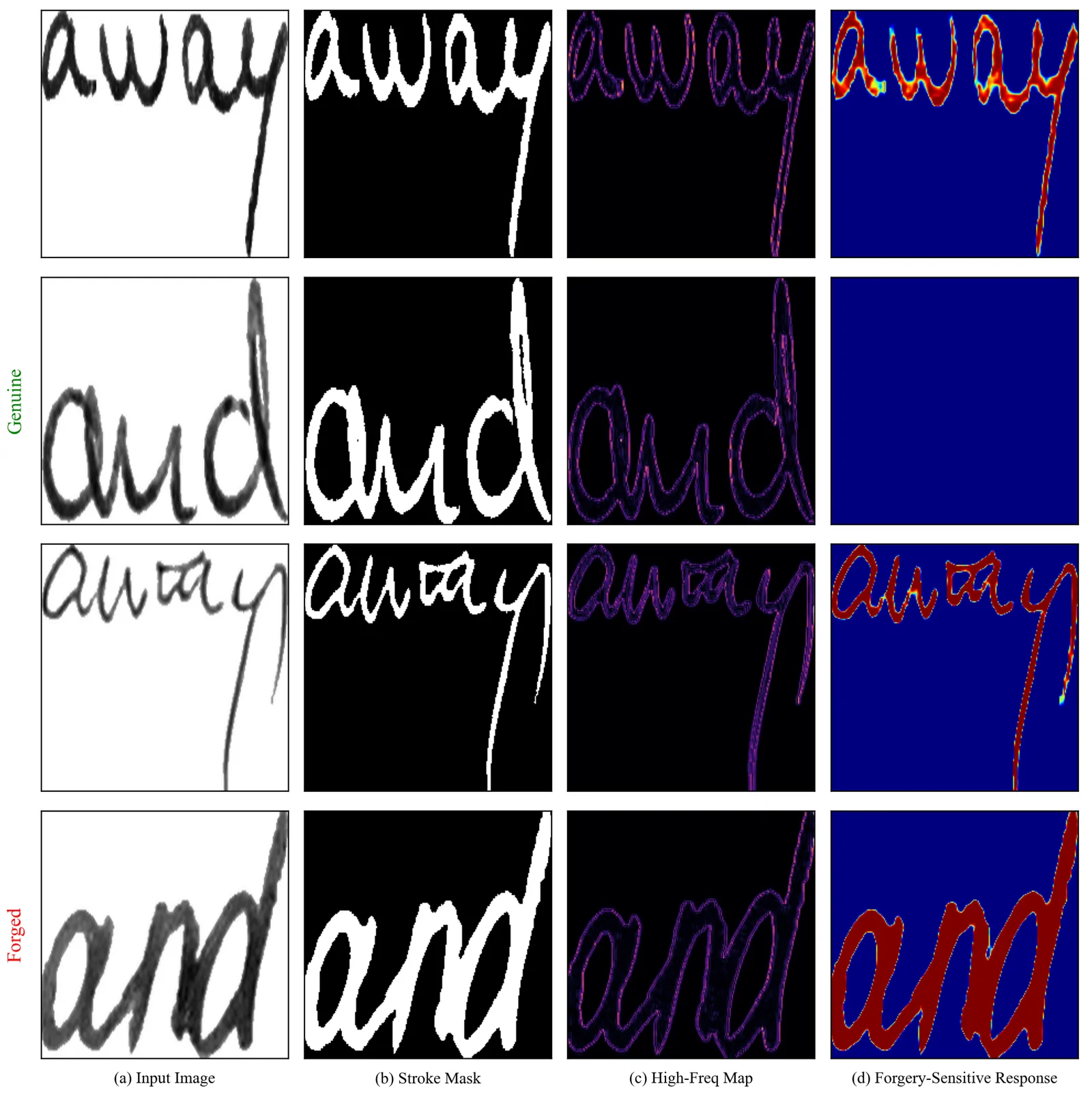
Qualitative comparison of forgery-sensitive stroke-region response maps between genuine and forged handwriting samples. Rows 1–2: genuine IAM samples. Rows 3–4: WordStylist-generated forged samples. All samples are correctly classified by MSF-DiffDet. Columns: (**a**) input image, (**b**) fixed-threshold stroke mask, (**c**) Laplacian-based high-frequency map, and (**d**) forgery-sensitive stroke-region response map. All response maps use the same color scale ([0,1]). The first genuine sample exhibits moderate activation on a subset of stroke segments, reflecting the intrinsic difficulty of this particular sample; the second genuine sample exhibits a near-zero response. Both forged samples exhibit strong and spatially concentrated responses. The displayed samples are individually selected for visualization and are not intended to reproduce the group means in Table 7. In particular, the first genuine sample exhibits moderate activation on a subset of stroke segments, which is consistent with the residual standard deviation of the genuine group in Table 7. The response maps are produced by a weakly supervised localization branch and should not be interpreted as pixel-level artifact ground-truth segmentation.

**Figure A2:**
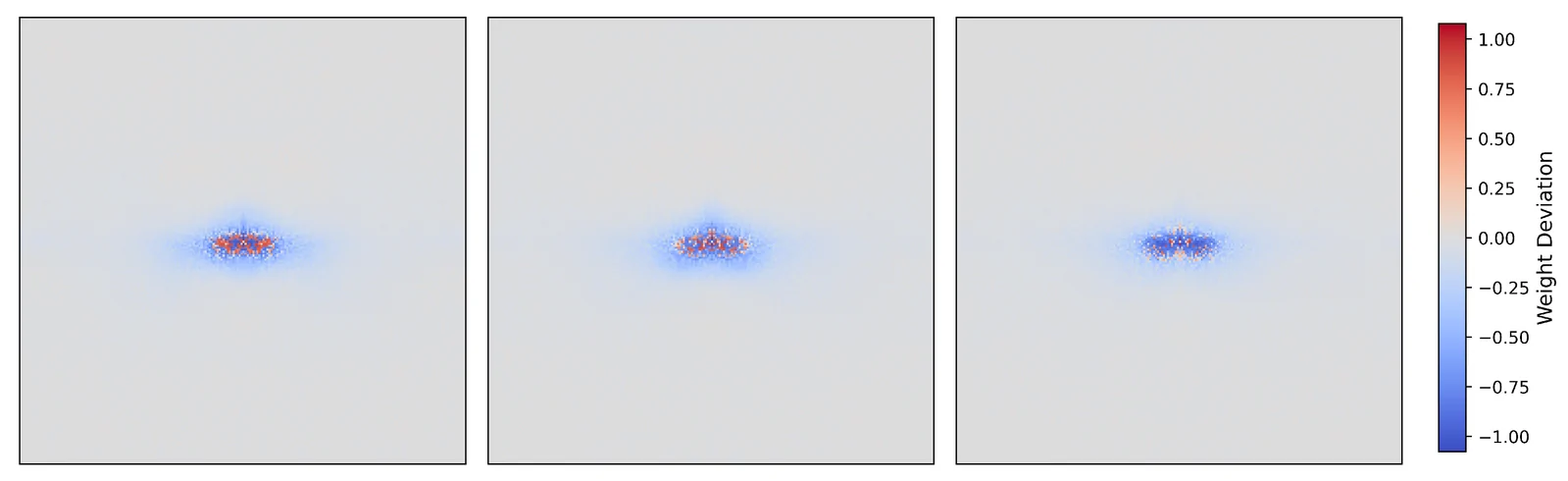
Visualization of the learned LFAD frequency masks as weight deviations from the uniform initialization (w−1.0). Left, middle, and right panels correspond to $M_{1}$, $M_{2}$, and $M_{3}$, respectively. Red regions denote frequencies that are amplified by the mask, while blue regions denote frequencies that are suppressed. The masks learn to adaptively allocate weights in the central frequency bands rather than applying hard-coded bandpass filters, capturing the most discriminative spectral traces for diffusion-generated handwriting detection.

**Figure A3:**
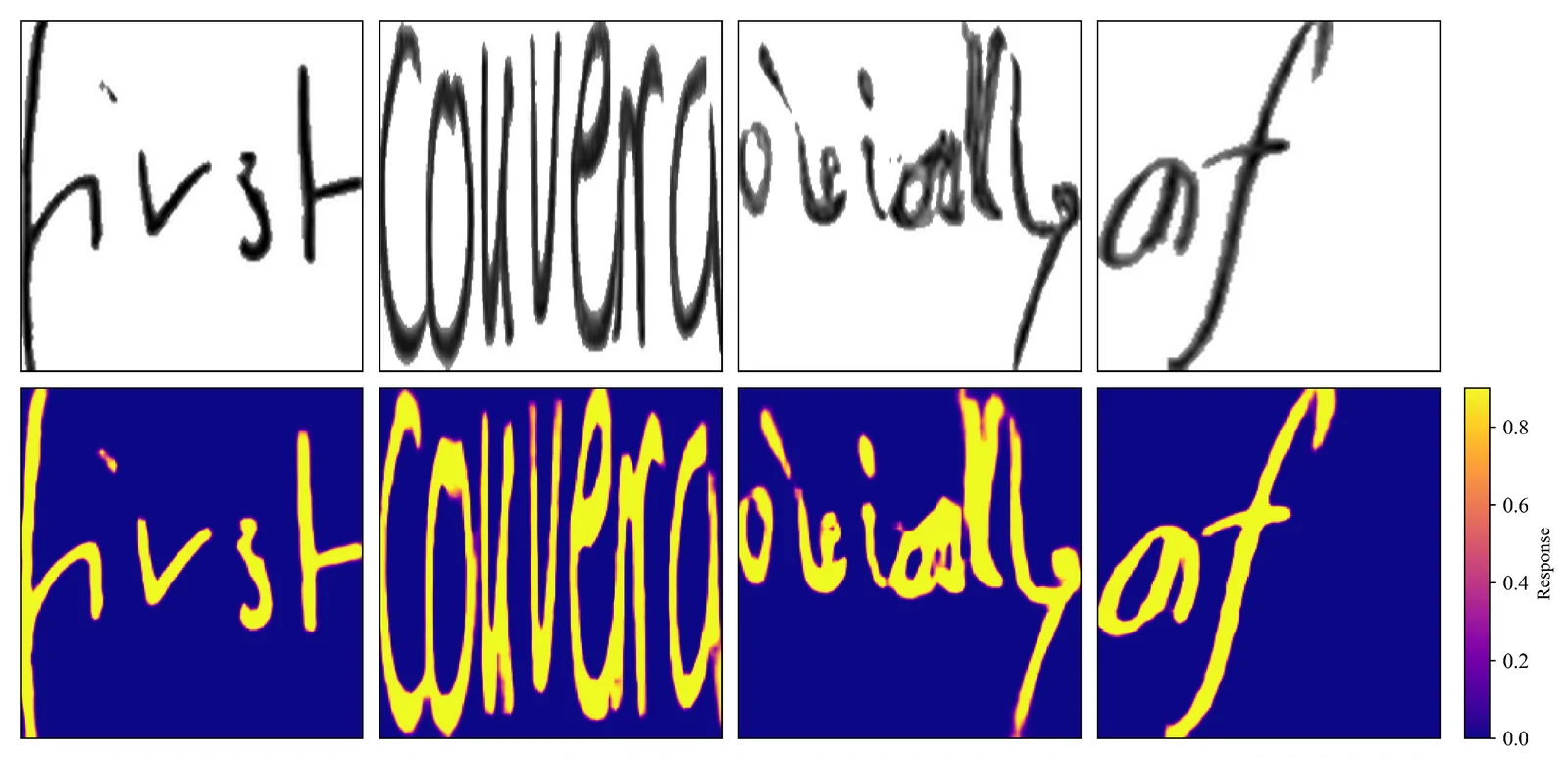
Response maps of four correctly classified forged samples sharing the same image label. Top row: input images. Bottom row: predicted response maps under a common color scale ([0,0.9]). All four samples are forged and correctly classified with high confidence ($p_{\mathrm{forged}}>0.99$). Although they share the same image label, the high-response regions (yellow) are distributed non-uniformly, localizing specific boundary irregularities rather than uniformly covering the entire stroke. This sample-specific spatial variation indicates that the response map encodes localized forgery evidence beyond a simple label-conditioned stroke mask.

Qualitative visualizations of the response maps for genuine and forged samples are provided in Appendix Figure A1. The visualizations are consistent with the quantitative comparison in Table 7: genuine samples exhibit weaker and more diffuse responses, whereas forged samples exhibit stronger and more spatially concentrated responses. We note that one displayed genuine sample shows moderate activation on a subset of stroke segments, which reflects the intrinsic difficulty of samples near the decision boundary and is consistent with the residual standard deviation of the genuine group in Table 7.

These results, together with the background-only control experiment in Section 4.4.3, provide complementary evidence that the image-level decision relies on stroke-containing regions and that the localization response discriminates genuine from forged strokes rather than simply performing stroke segmentation. As noted in Section 4.4.2, this comparison cannot fully rule out the possibility that the discriminative signal originates from general stroke appearance rather than diffusion-specific microscopic artifacts. The gold-standard validation would require mixed-patch test samples with manually annotated artifact ground truth, which we identify as future work.

### 4.5. Robustness Evaluation

Forensic images can often be subject to lossy compression, additive noise, optical blur, and resolution loss during acquisition and transmission. To test model robustness under non-ideal conditions, we used four common types of distortions on Test Set A: JPEG compression, Gaussian noise, Gaussian blur, and downsampling. For each distortion type, multiple severity levels were evaluated. Distortions were applied to the original test images before the same preprocessing pipeline, unless otherwise specified.

Table 8 summarizes the results under common image distortions. MSF-DiffDet achieves the highest accuracy at QF=90 (93.12%) and remains competitive at QF=50 (89.47%) with an EER of 18.62%. Under Gaussian noise, it shows particularly strong stability: its accuracy only drops from 91.88% at σ=5 to 90.84% at σ=10, with the EER increasing slightly from 12.67% to 15.88%. These results indicate that the proposed detector effectively exploits a mixture of fragile high-frequency stroke-boundary cues and relatively persistent spectral statistics and stroke-level structural information.

Gaussian blur has a much stronger negative impact on detection performance. Under moderate blur, CNNDetection achieves 78.21% accuracy, outperforming MSF-DiffDet (63.72%). Under severe blur, all eight compared methods degrade substantially, with EER values ranging approximately 40–50%, and MSF-DiffDet drops to 52.06%. This is because blur directly suppresses the fine high-frequency stroke-boundary cues that MixBlock relies on for cross-domain alignment. Under our unified protocol, no blur augmentation is used for any model; CNNDetection’s stronger blur robustness thus reflects its architecture and original training prior. Removing fine stroke boundaries and local frequency variations therefore remains a major challenge for the proposed model.

Under 0.5× downsampling, CNNDetection achieves the highest accuracy (85.14%), while MSF-DiffDet achieves the lowest EER (16.33%) despite a moderate accuracy of 77.62%. This discrepancy reflects a shift in the decision threshold after resolution reduction, rather than a genuine degradation in ranking capability. Overall, MSF-DiffDet exhibits good robustness against common digital-domain degradations including JPEG compression and additive Gaussian noise. Nevertheless, the model remains vulnerable to severe Gaussian blur and heavy resolution reduction via downsampling. Severe blur suppresses fine stroke-boundary high-frequency cues that the spatial-frequency interaction mechanism relies on, leading to a substantial performance drop, and several competing baselines can outperform our method under these harsh conditions. Real-world physical distortions such as print-and-rescanning, mobile-camera capture, and contrast adjustment are left for future investigation.

**Figure 5:**
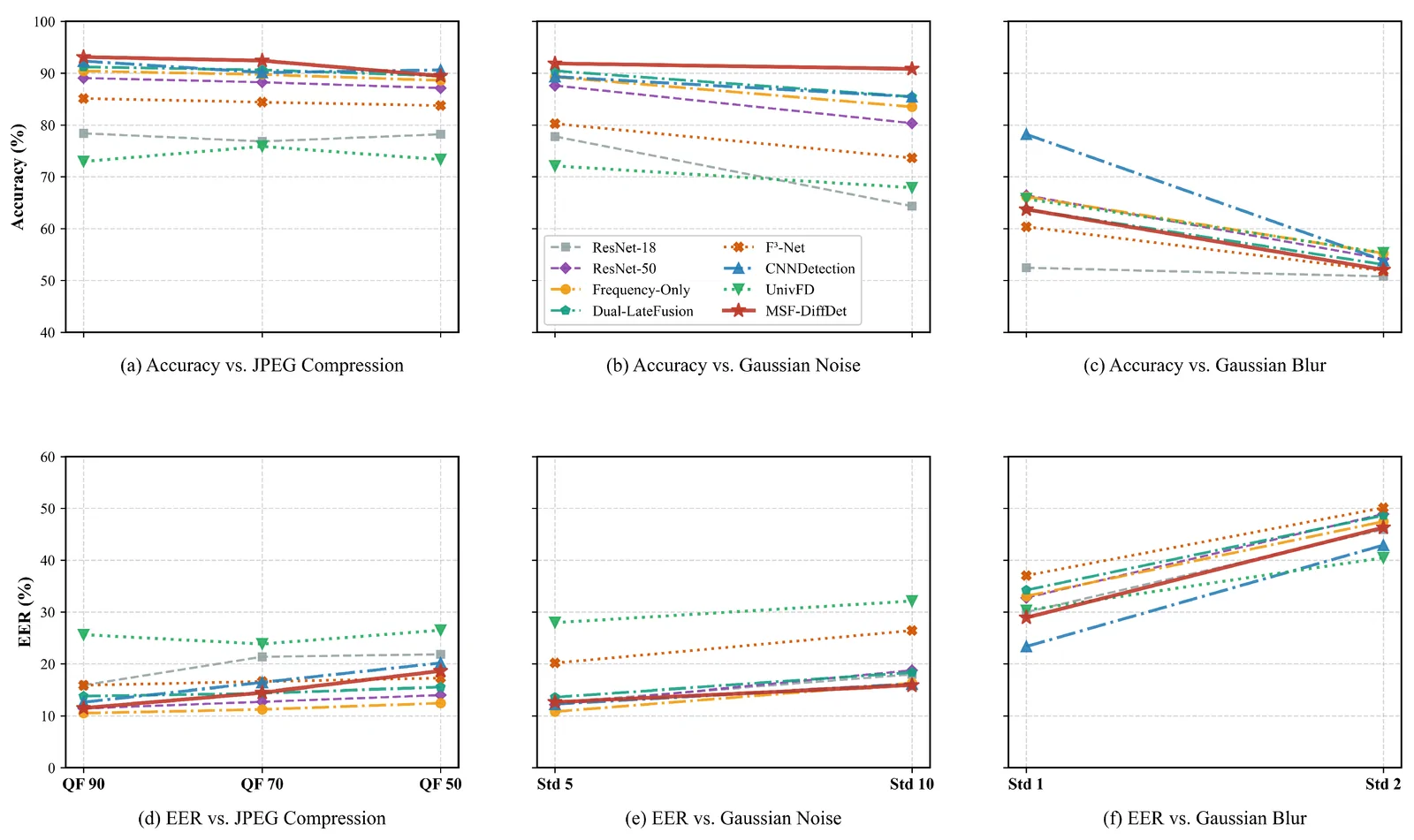
Accuracy and EER degradation curves under JPEG compression, Gaussian noise, and Gaussian blur. The downsampling results are reported in Table 8.

Figure 5 visualizes the degradation trends for JPEG compression, Gaussian noise, and Gaussian blur at different severity levels. The top row shows accuracy decay and the bottom row shows EER growth. JPEG compression and Gaussian noise lead to gradual performance degradation, whereas Gaussian blur causes a sharp performance drop across all models. This suggests that the proposed detector tolerates partial disturbance but is particularly sensitive to the loss of stroke boundary details.

We additionally evaluate model robustness under software-simulated print-and-scan degradation. This simulation applies a pipeline of halftone screening, additive scan noise, slight geometric distortion, paper-texture overlay, and illumination gradient to Test Set A. We emphasize that this is a software simulation and does not replace real hardware print-and-scan or mobile-camera acquisition. The results are reported in Table 9. Under the simulated print-scan artifacts, all compared models experience noticeable performance degradation, and MSF-DiffDet achieves 89.51% accuracy and 93.82% AUC, retaining a relative advantage in AUC over most baselines while remaining within 0.88 percentage points of CNNDetection in accuracy. Under severe degradation, fixed-threshold accuracy can collapse although AUC remains informative. A detailed calibration and operating-point analysis is provided in Section 4.6. This indicates that physical-optical document distortion remains a challenging open problem for diffusion-handwriting forgery detection. Real print-and-scan and mobile-camera datasets are designated as future work.

We also report computational efficiency. Appendix A Table A2 summarizes parameters, GFLOPs, GPU memory and latency, and additionally reports CPU-side inference latency and peak CPU memory for all compared models, measured on an Intel Core i9-10900K CPU using PyTorch with 10 CPU threads. MSF-DiffDet requires 301.46 ms per image and 975.2 MB of peak CPU memory. This is considerably heavier than lightweight spatial baselines such as ResNet-18 (15.16 ms, 526.5 MB) or ResNet-50 (37.00 ms, 623.7 MB), but it remains within a practically deployable range and is substantially lighter than the CLIP-based UnivFD baseline, which requires 428.41 ms and 2763.8 MB. The CPU overhead of MSF-DiffDet is consistent with its dual-stream architecture with learnable frequency decomposition and multi-scale cross-domain interaction.

### 4.6. Generalization Evaluation

Whereas the previous subsection addresses score shifts induced by image-level degradations, this section evaluates behavior under generator and script shifts. Zero-shot cross-domain evaluation provides a challenging setting for assessing whether a detector overfits to generator-specific artifacts. To evaluate the models’ generalization capability, this experiment is set up with three test environments featuring different distributions. All models are trained only on Dataset A. They are then evaluated without fine-tuning on Test Set A, the cross-generator Test Set B, and the joint-shift Test Set C. Note that Test Set C corresponds to multiple concurrent distribution shifts rather than pure cross-script generalization. The generalization performance comparison of models in cross-domain scenarios is presented in Table 10.

Table 10 shows a marked decline from intra-domain to cross-domain evaluation. On Test Set B, ResNet-18 achieves an AUC of 81.18% but only 50.14% accuracy at the default threshold, indicating a substantial shift in its output scores. MSF-DiffDet retains 89.16% accuracy, with an AUC of 92.73% and an EER of 10.90%. Notably, while UnivFD achieves a slightly higher AUC of 96.27% on this specific WordStylist → DiffBrush direction, its fixed-threshold accuracy drops to 70.82%, indicating a severe probability-distribution shift. To comprehensively evaluate unseen-generator generalization, we further conduct the reverse leave-one-generator-out (LOGO) experiment (DiffBrush→WordStylist)in Appendix A.8. As shown in Table A7, the baselines collapse in this reverse direction (UnivFD AUC 56.24%, Frequency-Only AUC 77.90%), whereas MSF-DiffDet maintains a robust AUC of 84.74%. This demonstrates that MSF-DiffDet provides a more balanced and robust cross-generator transfer across different transfer directions.

We emphasize that Test Set B shares the same IAM writing domain as the training distribution while using a different generator. Since both genuine and forged images in Test Set B originate from IAM, a detector that relies on dataset-specific characteristics such as scanning artifacts or paper texture would be unable to separate them. The strong performance of MSF-DiffDet on Test Set B therefore provides direct evidence that the model captures generation-process artifacts that transfer across generators, rather than memorizing dataset-specific statistics. The degradation on Test Set C, where the dataset, script, and generator all change simultaneously, is consistent with this interpretation.

The ranking of the methods depends on the evaluation criterion. On Test Set B, MSF-DiffDet achieves slightly higher fixed-threshold accuracy than CNNDetection and also performs better in terms of AUC and EER. UnivFD obtains the highest AUC and the lowest EER, but its fixed-threshold accuracy is much lower. Thus, UnivFD preserves strong class-ranking ability after generator shift, although its default threshold does not transfer well. More generally, the disagreement between accuracy and threshold-independent metrics reveals considerable calibration shifts across generators.

As summarized in Table 2, Test Set C varies multiple factors simultaneously and is therefore not a controlled cross-script evaluation. Its results provide evidence for robustness under joint-domain shift rather than a controlled evaluation of script-level transfer. All compared methods show substantial degradation under this combined setting. MSF-DiffDet achieves 64.88% accuracy, 65.91% AUC, and 35.20% EER, obtaining the best overall performance among the compared methods on this test set. Nevertheless, the remaining performance gap indicates that generalization under multiple concurrent distribution shifts remains limited.

We note that Test Set B serves as a single-factor control for generator shift, since it shares the same Latin script and IAM dataset family as the training data and differs only in the generative model and writer pool. Test Set B therefore provides a more isolated assessment of cross-generator transfer than Test Set C. Together, the two test sets allow the effect of a single-factor shift (Test B) to be compared with the effect of multiple simultaneous shifts (Test C).

We further analyze probability calibration, threshold behavior, and prediction uncertainty under domain shift. The Expected Calibration Error (ECE) with 10 equal-width bins is used to evaluate probabilistic calibration. Following common forensic practice, we select the decision threshold $\tau_{val}$ at the EER operating point on the held-out validation set and apply this fixed threshold to Test Sets A, B, and C. Prediction confidence is approximated by the maximum softmax output probability as a lightweight per-sample uncertainty proxy. Full numerical results are summarized in Table A6 of Appendix A.

The results reveal three consistent trends. First, the ECE increases monotonically from 0.38% on Test Set A to 4.00% on Test Set B and 25.12% on Test Set C. Test Set A exhibits excellent in-distribution calibration, with an accuracy of approximately 93.5% under the fixed validation threshold, which is consistent with the intra-domain result reported in Table 3. As the domain shift grows, however, the model becomes progressively more miscalibrated, consistent with the well-known tendency of neural classifiers to be overconfident on out-of-distribution inputs. Second, the fixed validation-set threshold produces increasingly asymmetric operating points across domains. On Test Set B, the FPR and FNR are 13.85% and 7.83%, respectively, indicating a mild imbalance. On Test Set C, the FNR reaches 40.52% while the FPR is 29.72%, meaning that the model becomes biased toward predicting “genuine” and thus misses a large fraction of forged samples. Note that these FPR/FNR values are computed at the fixed validation threshold $\tau_{val}$, where FPR≠FNR; they therefore cannot be averaged to estimate the threshold-independent EER reported in Table 3 and Table 10. From a forensic deployment perspective, this direction of error (missed forgeries) is more consequential than a false alarm, and it motivates dataset-specific threshold adaptation or adaptive operating-point selection in real systems. Third, the gap between the mean confidence of correctly and incorrectly classified samples shrinks monotonically from 0.212 on Test Set A to 0.123 on Test Set B and 0.059 on Test Set C. This shows that the simple softmax-max confidence proxy becomes less discriminative under stronger domain shift, and that a more rigorous uncertainty estimator is needed for reliable forensic decision support. A per-sample uncertainty estimator based on Monte Carlo Dropout would require network modification and full multi-seed retraining, which we identify as future work.

Finally, we note that the interpolation control experiment in Appendix A.9 (Table A8) shows that the AUC varies within 0.17 percentage points across four resize interpolation modes, confirming that the detector is not driven by interpolation-specific artifacts.

To isolate the effect of generator shift from the effects of script and dataset shift, we conduct a strict LOGO experiment (Section 4.1.2). MSF-DiffDet achieves the highest cross-generator AUC (84.74% ± 0.98%) among the four compared models, followed by ResNet-50 (Spatial-Only) (81.68% ± 1.22%), Frequency-Only (77.90% ± 1.81%), and UnivFD (56.24% ± 0.13%). The ranking differs from the in-generator evaluation: in the LOGO setting, the purely spatial ResNet-50 outperforms the purely frequency-domain variant, suggesting that spatial texture features provide the primary cross-generator transfer signal while frequency-domain features partially overfit to generator-specific spectral artifacts. The dual-stream MSF-DiffDet yields the best cross-generator performance, supporting the design of the spatial-frequency interaction. Fixed-threshold metrics show a consistent conservative bias across all models (precision 0.99, recall 0.06–0.09), reflecting a probability-distribution shift rather than a failure of ranking capability. The full results are reported in Appendix A.8 (Table A7).

### 4.7. Ablation Study

To evaluate the effectiveness of the proposed components, we conducted a progressive ablation study by incrementally extending a spatial-only model. M1 uses the same basic encoder–decoder topology as MSF-DiffDet but excludes LFAD, LFS, MixBlock, and AF-Skip. It should be distinguished from the standalone ResNet-50-S classifier reported in Table 3 because M1 retains the encoder–decoder structure of the proposed framework.

Starting from M1, M2 introduces LFAD to extract learnable frequency-domain features. M3 further incorporates LFS to characterize local statistical variations in the frequency representations. M4 adds MixBlock to enable hierarchical interaction between the spatial and frequency features. Finally, M5 introduces AF-Skip for adaptive skip-feature fusion and corresponds to the complete MSF-DiffDet model. In the variants without MixBlock, the available spatial and frequency features are fused using element-wise addition. All variants were retrained and evaluated using the same data splits, hyperparameters, and training strategy. The results are presented in Table 11.

The ablation results in Table 11 reveal how each component contributes to overall performance, based on averaged results across three independent training seeds with standard deviations reported for all metrics. The spatial-only baseline M1 achieves 88.31% accuracy, 91.16% AUC, and 17.04% EER. LFAD improves fixed-threshold classification accuracy by +1.87% in Acc, while LFS enhances discriminative score separation, yielding further improvements of +0.79% in Acc and +1.57% in AUC. The two frequency-oriented modules collectively enrich spectral representations, with LFS capturing local statistical variations within these features, leading to a combined accuracy improvement of +2.66% and an AUC increase of +2.15%.

MixBlock provides a substantial accuracy gain of +1.61% and an AUC improvement of +1.41% over M3, demonstrating that explicit interaction between spatial and frequency features is more effective than treating the two branches separately. AF-Skip yields a moderate accuracy gain of +0.91% while providing the largest EER reduction of 2.51 percentage points among all components, indicating that adaptive decoder fusion primarily improves class separation rather than overall classification accuracy.

The full model (M5) achieves 93.49% accuracy, 96.30% AUC, and 10.45% EER, corresponding to improvements of +5.18% in accuracy, +5.14% in AUC, and a reduction of 6.59 percentage points in EER relative to M1 (Table 3). These performance gains are distributed across frequency modeling (LFAD+LFS: +2.66% Acc), cross-domain interaction (MixBlock: +1.61% Acc), and adaptive decoder fusion (AF-Skip: +0.91% Acc), instead of being dominated by a single component. All reported gains are substantially larger than the corresponding inter-seed standard deviations, confirming their statistical stability.

To verify that each component contributes a statistically reliable gain, we applied the same paired bootstrap and McNemar tests to adjacent ablation variants. The results are reported in Table A4 of Appendix A. All four adjacent transitions exhibit statistically significant AUC improvements, with the 95% bootstrap confidence intervals entirely above zero. For fixed-threshold accuracy, the transitions associated with MixBlock (M3 to M4) and LFS (M2 to M3) are statistically significant at the 0.001 and 0.05 levels, respectively. In contrast, the LFAD-only transition (M1 to M2) and the AF-Skip transition (M4 to M5) do not reach conventional significance (p=0.092 and p=0.069). This is consistent with the interpretation that LFAD and AF-Skip primarily enhance the discriminative score separation, which is captured by AUC and EER, whereas MixBlock and LFS also contribute to fixed-threshold classification. These non-significant accuracy transitions do not weaken the ablation conclusion: the threshold-independent metrics show consistent and highly significant gains at every step, and the total AUC improvement from M1 to M5 is +0.0514, which is about 12 times the inter-seed standard deviation of ±0.0044.

### 4.8. Failure Case Analysis and Limitations

**Figure 6:**
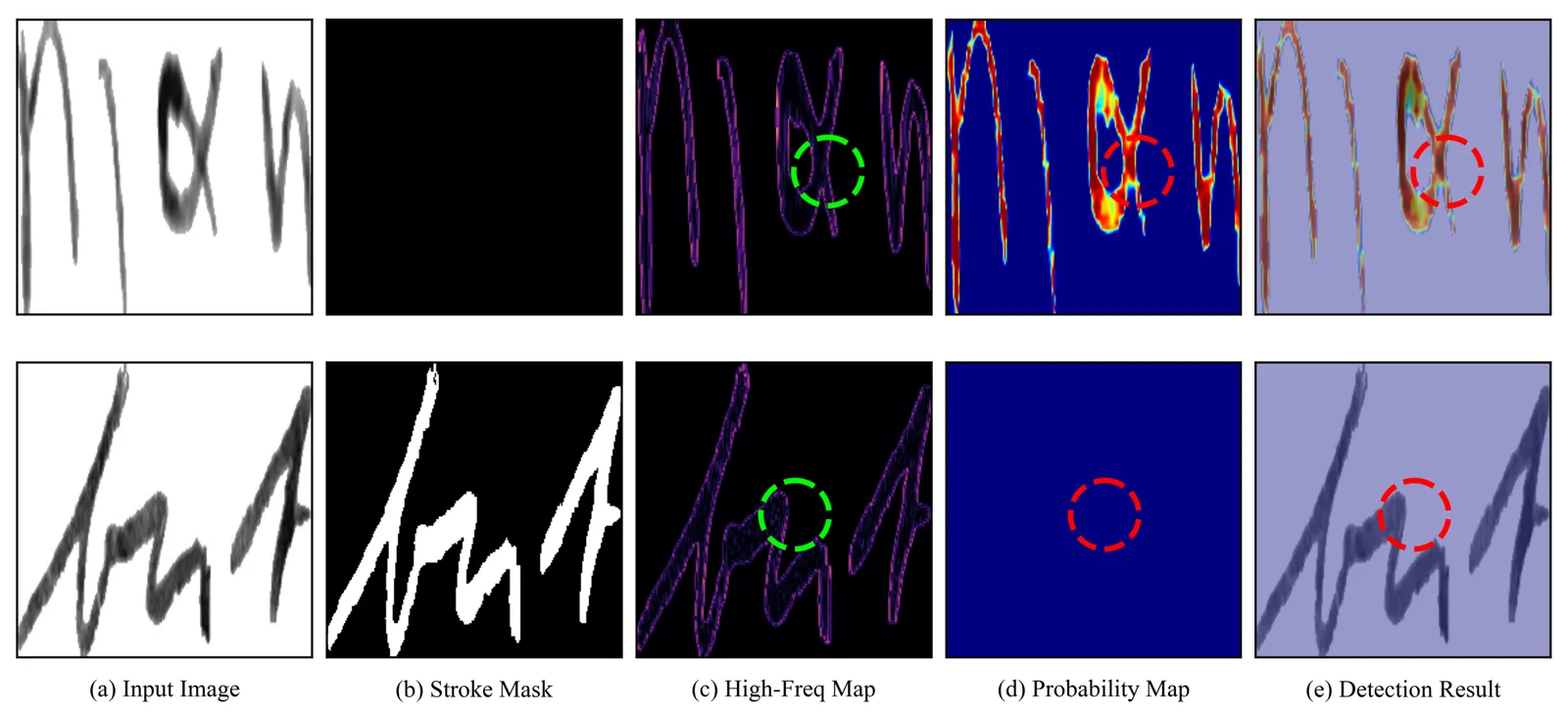
Representative failure cases of MSF-DiffDet. From left to right: input image, weak stroke-region supervision target, high-frequency response, predicted forgery-sensitive stroke-region response, and detection result. The dashed green and red circles highlight the regions focused on by the high-frequency features and the predicted forgery-sensitive responses, respectively.

Although MSF-DiffDet is strong overall, it still produces errors under some challenging conditions. Figure 6 shows two failure cases from Test Set A.

The upper panel false positives: the sample with short word, sparse ink coverage and mild scanning noise is wrongly classified as generated. High-frequency and localization responses focus on isolated stroke fragments, which have fine irregularities similar to those found in diffusion-generated strokes. The difference between real and synthesized samples is narrow with only a small background content.

The lower panel shows false negatives: the generated sample has smoother strokes and weaker boundary artifacts than typical WordStylist output and the high-frequency response is diffuse rather than spatially localized. The classification head returns a low forged-class score, and the localization branch focuses only on stroke regions in the generated sample. This behavior is consistent with cases where the generator outputs lie closer to the natural stroke distribution.

All of these cases point to three practical problems. First, the detector is somewhat sensitive to stroke density, and local boundary structure and content-sparse inputs are difficult to obtain. Second, the blur, aggressive downsampling or heavy ink degradation can reduce fine-grained signals that frequency modules rely on. Third, the localization branch is trained under weak stroke-region supervision and is not calibrated against pixel-level artifact annotations.

Mitigating these issues will require physically motivated degradation augmentation, foreground-aware feature aggregation for sparse inputs, broader generator coverage during training, and uncertainty-aware decision rules that can distinguish confident predictions from borderline cases.

## 5. Conclusions

This paper presented MSF-DiffDet, a U-shaped spatial-frequency interactive network for detecting diffusion-generated handwriting. The model integrates learnable frequency decomposition, local spectral statistics, hierarchical spatial-frequency interaction, and adaptive skip fusion within a single encoder–decoder framework. A weakly supervised localization branch is trained jointly with the image-level classifier and produces forgery-sensitive stroke-region response maps.

On the writer-disjoint intra-domain test set, MSF-DiffDet achieves 93.49% accuracy, 96.30% AUC, and 10.45% EER, and remains competitive under JPEG compression, additive noise, moderate blur, and resolution reduction. On the unseen DiffBrush generator, it retains 89.16% accuracy. Furthermore, in strict leave-one-generator-out evaluations across both transfer directions, it achieves the highest cross-generator AUC of 84.74%, demonstrating that spatial features provide the primary transferable cue. Under the joint multi-factor domain shift, the model still provides the best overall result among the compared methods, although absolute performance is limited. Finally, the ablation study shows that the accuracy and AUC gains are distributed across frequency modeling, cross-domain interaction, and adaptive fusion rather than concentrated in a single component.

Three complementary control experiments were conducted to characterize the forgery-sensitive stroke-region response. The deletion-based analysis shows that high-response regions are coupled to the classification output, although the evidence is correlational rather than a complete causal decomposition. The background-only control experiment shows that the image-level decision relies primarily on stroke-containing regions rather than background texture alone. The pure stroke-segmentation baseline shows that the localization branch does not merely highlight handwriting strokes: a pure stroke segmenter produces nearly identical responses on genuine and forged strokes with a separation of only −0.0003, whereas MSF-DiffDet achieves a separation of +0.9591, corresponding to an effect-size ratio of approximately 109. In addition, the calibration analysis shows that the expected calibration error grows from 0.38% under the in-domain setting to 25.12% under the joint multi-factor shift, and that the fixed validation-set threshold yields a strongly asymmetric operating point with a 40.52% false negative rate on the joint-shift test set. These results highlight that threshold adaptation and uncertainty-aware decision rules are important for forensic deployment.

The detector is sensitive to severe blur and sparse foreground content. It has not been studied under heavier ink degradation, and cross-script transfer remains insufficiently investigated. In addition, the weak stroke-mask supervision makes it difficult to fully disentangle stroke-related responses from responses originating from diffusion-specific microscopic artifacts; this remains an open question.

Future work will proceed along four directions. First, mixed-patch test samples with manually annotated diffusion artifacts will be constructed to further disentangle stroke-related responses from artifact-specific ones. Second, real physically acquired datasets, including print-and-scan and mobile-camera captured handwriting specimens, will be constructed to evaluate robustness under real optical-document degradation beyond software simulation. Third, a strictly controlled factorial benchmark that separates script shift, generator shift, and dataset-related acquisition shift will be developed; such a benchmark would require a unified generator framework trained or adapted under matched protocols for both Latin and Chinese handwriting, together with genuine-image datasets whose acquisition, preprocessing, resolution, and content distributions are controlled as closely as possible. Fourth, a direct quantitative comparison with reconstruction-based diffusion detectors such as DIRE and FIRE will be pursued on a handwriting-adapted diffusion model. 

## Figures and Tables

**Table 1:** Configuration and feature dimensions of the dual-branch encoder.

Network Node	Input Size	Output Size	Description
Spatial Entry	1×224×224	3×224×224	Initial visual feature extraction and spatial downsampling
LFAD	1×224×224	3×224×224	Frequency-aware decomposition (learnable spectral components)
Entry Conv	3×224×224	256×56×56	Initial downsampling
LFS Branch	256×56×56	256×56×56	Local variance computation for texture monotony capture
Mix stage1	256×56×56	512×28×28	Local texture extraction for initial cross-domain interaction
Mix stage2	512×28×28	1024×14×14	Structural feature extraction for spatial-frequency alignment
Mix stage3	1024×14×14	2048×7×7	Global semantic extraction for final logical calibration

**Table 2:** Factors that vary across the four evaluation settings. A checkmark indicates that the factor differs from the training distribution; a dash indicates that it is matched. Test Set B primarily isolates generator shift under the same Latin script and IAM dataset family. Test Set C changes multiple factors simultaneously and therefore represents a joint-domain rather than a cross-script evaluation. The LOGO setting isolates generator shift while keeping the script, dataset family, and preprocessing matched, and it additionally enforces writer-disjoint splits on all three sides.

Factor	Test A	Test B	Test C	LOGO
Handwriting script	—	—	✔	—
Genuine-image source	—	—	✔	—
Writer pool	—	✔	✔	✔
Generative model	—	✔	✔	✔
Acquisition/preprocessing	—	—	✔	—

**Table 3:** Performance comparison of baseline models on Test Set A. Because fixed-threshold accuracy can vary with the chosen decision threshold, the primary comparison metrics are the threshold-independent AUC and EER; fixed-threshold Accuracy, Precision, Recall, and F1 are reported for completeness. All results are averaged over three independent random seeds, with standard deviations reported. The localization-loss weight λ was selected on the validation split (see Appendix A Table A1). The downward arrow (↓) denotes that lower values indicate better performance.

Method	Acc (%)	Precision (%)	Recall (%)	F1 (%)	AUC (%)	EER (%) ↓
ResNet-18	89.85 ± 0.58	88.95 ± 0.64	90.76 ± 0.71	89.85 ± 0.59	93.90 ± 0.47	14.19 ± 0.83
ResNet-50 (Spatial-Only)	92.76 ± 0.34	92.55 ± 0.41	92.90 ± 0.46	92.72 ± 0.37	95.71 ± 0.27	11.18 ± 0.55
Frequency-Only	91.69 ± 0.61	91.42 ± 0.69	91.95 ± 0.74	91.68 ± 0.62	94.96 ± 0.52	12.35 ± 0.89
Dual-LateFusion	92.64 ± 0.43	92.41 ± 0.49	92.88 ± 0.53	92.64 ± 0.44	95.58 ± 0.36	11.32 ± 0.62
Fixed-FFT Dual-Branch	91.35 ± 0.27	91.67 ± 0.25	91.02 ± 0.27	91.35 ± 0.26	93.96 ± 0.27	12.59 ± 0.28
F^3^-Net	91.52 ± 0.72	91.28 ± 0.78	91.77 ± 0.85	91.52 ± 0.74	94.83 ± 0.59	12.49 ± 0.97
CNNDetection	92.04 ± 0.39	91.67 ± 0.44	92.41 ± 0.48	92.04 ± 0.40	95.22 ± 0.29	11.76 ± 0.58
UnivFD	78.80 ± 0.94	79.70 ± 1.02	77.91 ± 1.08	78.80 ± 0.96	86.53 ± 0.81	22.26 ± 1.36
MSF-DiffDet (Ours)	93.49 ± 0.35	93.32 ± 0.33	93.66 ± 0.37	93.49 ± 0.29	96.30 ± 0.44	10.45 ± 0.22

**Table 4:** Global normalized deletion AUCs and image-wise paired differences over 100 correctly classified forged images. Lower AUC indicates a stronger deletion effect under the global pixel-replacement protocol.

AUCs and Paired Differences	Mean	95% Bootstrap CI
Top-ranked AUC	0.1458	[0.1328, 0.1604]
Random AUC	0.4813	[0.4362, 0.5276]
Bottom-ranked AUC	1.0000	[1.0000, 1.0000]
Random (Top-ranked)	0.3355	[0.2920, 0.3796]
Bottom-ranked (Top-ranked)	0.8542	[0.8396, 0.8672]

**Table 5:** Foreground-constrained normalized deletion AUCs and image-wise paired differences over the same 100 correctly classified forged images. Random deletion was independently repeated 20 times per image.

AUCs and Paired Differences	Mean	95% Bootstrap CI
Top-ranked AUC	0.3645	[0.3229, 0.4078]
Random AUC	0.4782	[0.4328, 0.5244]
Bottom-ranked AUC	0.3991	[0.3713, 0.4287]
Random (Top-ranked)	0.1137	[0.0670, 0.1614]
Bottom-ranked (Top-ranked)	0.0346	[−0.0035, 0.0723]

**Table 6:** Background-only control inference results over the same 100 correctly-classified forged images. All probabilities are image-level forged-class probabilities. Pairwise *t*-tests are paired at the image level and compared against G1 (original). Lower values indicate a stronger contribution of the removed region to the forgery decision.

Condition	Mean ± Std	Drop vs. G1	Paired *t*-Test *p*
G1 Original	0.9999±0.0004	—	—
G2 Background-only	0.8096±0.1795	0.1904	$5.24\times 10^{-18}$
G3 Stroke-only	0.9566±0.1747	0.0433	$1.47\times 10^{-2}$
G4 Random-mask	0.2529±0.3659	0.7470	$2.87\times 10^{-37}$

**Table 7:** Comparison of mean stroke-region responses between a pure stroke-segmentation baseline and the MSF-DiffDet localization branch. Responses are averaged over fixed-threshold stroke pixels of correctly-classified Test Set A samples. The segmentation baseline is trained only on stroke masks, without any image-level genuineness label. Separation denotes the difference between forged and genuine mean responses. Cohen’s *d* is computed for forged vs. genuine responses; positive values indicate higher response on forged strokes.

Method	Genuine	Forged	Separation	Cohen’s *d*
Stroke-seg baseline	0.9980±0.0008	0.9977±0.0016	−0.0003	−0.25
MSF-DiffDet	0.0027±0.0332	0.9618±0.0372	+0.9591	+27.21

**Table 8:** Full robustness results of all eight models under various degradation conditions. Metrics are reported as mean ± standard deviation (%) over three independent training seeds. For each model, the first row reports Acc and the second row reports EER (lower is better).

Method	Metric	JPEG-QF90	JPEG-QF70	JPEG-QF50	Noise-σ5	Noise-σ10	Blur-σ1	Blur-σ2	Scale-0.5×
ResNet-18	Acc	78.40 ± 0.27	76.84 ± 0.43	78.21 ± 0.75	77.79 ± 1.15	64.34 ± 1.50	52.45 ± 0.12	50.78 ± 0.35	67.05 ± 1.07
	EER↓	15.87 ± 0.36	21.35 ± 0.32	21.83 ± 0.43	12.61 ± 0.55	17.96 ± 0.70	29.97 ± 0.56	45.94 ± 0.95	25.82 ± 0.51
ResNet-50	Acc	89.07 ± 1.04	88.26 ± 0.55	87.14 ± 0.46	87.62 ± 1.13	80.35 ± 1.81	66.43 ± 1.66	54.16 ± 0.41	71.25 ± 1.00
(Spatial-Only)	EER↓	11.45 ± 0.25	12.73 ± 0.32	14.02 ± 0.44	12.17 ± 0.49	18.74 ± 0.56	32.75 ± 1.16	48.92 ± 5.63	28.41 ± 0.86
Frequency-Only	Acc	90.42 ± 0.05	89.75 ± 0.27	88.61 ± 0.10	89.27 ± 0.12	83.52 ± 0.30	66.18 ± 2.13	55.23 ± 1.17	75.63 ± 2.12
	EER↓	10.53 ± 0.05	11.26 ± 0.22	12.47 ± 0.14	10.84 ± 0.13	16.28 ± 0.34	33.06 ± 0.45	47.47 ± 1.08	22.74 ± 0.69
Dual-LateFusion	Acc	91.20 ± 0.02	90.63 ± 0.13	89.48 ± 0.13	90.46 ± 0.25	85.47 ± 0.40	63.74 ± 1.54	53.07 ± 1.30	72.38 ± 2.03
	EER↓	13.81 ± 0.03	14.38 ± 0.11	15.53 ± 0.16	13.62 ± 0.17	18.33 ± 0.26	34.22 ± 1.31	48.64 ± 1.19	25.16 ± 1.39
F^3^-Net	Acc	85.13 ± 1.03	84.41 ± 0.30	83.75 ± 0.14	80.26 ± 2.35	73.64 ± 3.13	60.37 ± 3.82	51.84 ± 0.78	70.42 ± 4.35
	EER↓	15.87 ± 0.49	16.58 ± 0.22	17.24 ± 0.11	20.15 ± 1.49	26.42 ± 2.18	37.04 ± 5.74	50.13 ± 2.29	27.83 ± 2.74
CNNDetection	Acc	92.35 ± 0.34	90.14 ± 0.40	90.62 ± 0.47	89.33 ± 1.24	85.46 ± 2.46	78.21 ± 1.61	53.88 ± 0.83	85.14 ± 1.31
	EER↓	12.66 ± 0.41	16.42 ± 0.38	20.17 ± 0.44	12.28 ± 0.53	16.13 ± 0.98	23.34 ± 1.13	42.91 ± 1.94	21.56 ± 0.84
UnivFD	Acc	72.96 ± 0.68	75.87 ± 0.72	73.34 ± 0.79	72.11 ± 2.01	67.92 ± 3.27	65.74 ± 2.34	55.33 ± 0.71	70.17 ± 2.09
	EER↓	25.61 ± 0.82	23.83 ± 0.77	26.48 ± 0.86	27.94 ± 1.22	32.11 ± 1.63	30.32 ± 2.18	40.46 ± 1.12	27.48 ± 1.37
MSF-DiffDet	Acc	93.12 ± 0.29	92.43 ± 0.28	89.47 ± 0.15	91.88 ± 0.15	90.84 ± 0.48	63.72 ± 1.20	52.06 ± 0.67	77.62 ± 2.40
(Ours)	EER↓	11.52 ± 0.31	14.48 ± 0.29	18.62 ± 0.19	12.67 ± 0.14	15.88 ± 0.62	28.91 ± 1.05	46.31 ± 3.57	16.33 ± 3.38

**Table 9:** Performance under software-simulated print-and-scan degradation on Test Set A. This simulation introduces halftone screening, additive scan noise, slight geometric distortion, paper-texture overlay, and illumination gradient. It is a software simulation and does not replace real physical print-and-scan or mobile-camera acquisition. All results are reported as mean ± standard deviation (%) over three independent training seeds where applicable.

Method	Acc (%)	AUC (%)	EER (%)↓
ResNet-18	52.35±1.15	87.94±0.38	20.42±0.42
ResNet-50 (Spatial-Only)	84.39±3.02	88.82±0.68	16.76±0.63
Frequency-Only	84.73±0.20	90.83±0.18	13.91±0.44
Dual-LateFusion	86.30±0.67	92.60±0.13	12.55±0.37
Fixed-FFT Dual-Branch	87.01±0.92	92.64±0.43	12.96±0.47
F^3^-Net	59.41±2.85	89.72±4.49	16.90±5.32
CNNDetection	90.39±0.88	92.80±0.41	11.76±0.45
UnivFD	63.42±1.21	68.99±0.87	36.16±0.94
MSF-DiffDet (Ours)	89.51±0.94	93.82±0.05	11.93±0.30

**Table 10:** Generalization performance comparison of models under cross-generator (Test Set B) and joint-shift (Test Set C) scenarios. All results are reported as mean ± standard deviation (%) over three independent training seeds. Results for Test Set A are reported in Table 3.

Method	Cross-Generator Test Set B			Joint-Shift Test Set C		
	Acc (%)	AUC (%)	EER (%)↓	Acc (%)	AUC (%)	EER (%)↓
ResNet-18	50.14 ± 2.50	81.18 ± 0.38	28.86 ± 0.54	50.13 ± 0.31	28.22 ± 1.56	66.88 ± 1.38
ResNet-50-S	65.52 ± 2.22	85.86 ± 2.40	22.80 ± 2.30	45.94 ± 1.60	31.30 ± 3.20	63.47 ± 3.10
Frequency-Only	84.33 ± 3.40	93.28 ± 3.58	14.35 ± 4.07	44.89 ± 3.02	39.37 ± 3.50	58.73 ± 3.20
Dual-LateFusion	88.59 ± 1.85	92.61 ± 0.83	11.31 ± 2.50	40.48 ± 1.25	33.71 ± 1.15	62.30 ± 1.27
F^3^-Net	71.23 ± 0.87	88.69 ± 0.42	17.57 ± 0.42	53.55 ± 0.51	46.73 ± 0.83	52.73 ± 0.77
CNNDetection	88.86 ± 0.33	90.71 ± 0.34	17.83 ± 0.38	45.22 ± 0.41	43.38 ± 0.23	54.24 ± 0.35
UnivFD	70.82 ± 0.39	96.27 ± 0.34	10.14 ± 0.31	52.41 ± 0.36	57.22 ± 0.26	46.63 ± 0.42
MSF-DiffDet (Ours)	89.16 ± 1.42	92.73 ± 0.70	10.90 ± 1.94	64.88 ± 2.56	65.91 ± 3.65	35.20 ± 2.80

**Table 11:** Progressive ablation results for the key components of MSF-DiffDet. All metrics are mean ± standard deviation (%) over three independent training seeds. ✔ denotes that the corresponding component is enabled, while “–” denotes that it is disabled.

Method	LFAD	LFS	MixBlock	AFSkip	Acc (%)	AUC (%)	EER (%)↓
M1: Baseline	–	–	–	–	88.31 ± 0.37	91.16 ± 0.29	17.04 ± 0.42
M2: +LFAD	✔	–	–	–	90.18 ± 0.32	91.74 ± 0.26	16.47 ± 0.38
M3: +LFAD+LFS	✔	✔	–	–	90.97 ± 0.35	93.31 ± 0.28	14.18 ± 0.41
M4: +LFAD+LFS+MixBlock	✔	✔	✔	–	92.58 ± 0.30	94.72 ± 0.24	12.96 ± 0.36
M5: Full MSF-DiffDet	✔	✔	✔	✔	93.49 ± 0.35	96.30 ± 0.44	10.45 ± 0.22

## Data Availability

The raw images are from the public datasets IAM and HWDB2.2 and cannot be redistributed. Writer-ID split lists (with sample identifiers), code, generation configurations, and trained model checkpoints will be released in a public GitHub repository upon publication (https://github.com/). The repository URL will be provided at that time.

## Appendix A

### Appendix A.1

The localization-loss weight λ in Equation (14) was selected via grid search over the values 1.0, 5.0, 10.0, 20.0, and 50.0, exclusively on the writer-disjoint validation split. Test Set A was never used for hyperparameter tuning. Table A1 reports the validation-set performance under each candidate λ.

**Table A1:** Validation-set performance under different candidate λ values.

λ	Val. Acc (%)	Val. AUC (%)	Val. EER (%)↓
1.0	91.37	94.48	13.24
5.0	93.61	96.22	10.38
10.0	92.84	95.63	11.19
20.0	92.15	95.07	11.96
50.0	90.68	93.81	14.12

The value λ=5.0 yields the best validation trade-off between classification accuracy and localization response quality and is therefore adopted for all final multi-seed experiments.

### Appendix A.2

Table A2 summarizes the computational complexity of all compared models. All metrics are measured on a single NVIDIA RTX 3090 GPU with batch size 1 and input size 1×224×224.

**Table A2:** Computational complexity comparison of all compared models. Metrics are evaluated at input size 1×224×224. Params: total parameters (M); GFLOPs: giga multiply-accumulate operations (MACs); GPU Mem: peak inference GPU memory (MB); GPU Inf.Time: average per-image inference latency (ms) on an NVIDIA RTX 3090 with batch size 1. CPU Inf.Time and CPU Peak Mem are measured on an Intel Core i9-10900K CPU (10 cores, 3.70 GHz) with PyTorch, using 10 CPU threads and no GPU acceleration. Latency is averaged over 500 forward passes after 50 warm-up passes.

Method	Params (M)	GFLOPs	GPU Mem (MB)	GPU Inf.Time (ms)	CPU Inf.Time (ms)	CPU Peak Mem (MB)
ResNet-18	11.18	1.824	105.5	2.49	15.16	526.5
ResNet-50 (Spatial-Only)	23.51	4.132	202.1	6.31	37.00	623.7
Frequency-Only	34.33	18.019	367.4	7.66	79.17	721.4
Dual-LateFusion	62.86	23.415	652.6	15.44	127.30	948.8
Fixed-FFT Dual-Branch	61.75	22.979	590.0	15.10	270.45	978.1
F^3^-Net	44.01	9.792	375.0	14.12	92.56	742.6
CNNDetection	23.51	4.132	242.4	6.45	35.53	797.9
UnivFD	202.05	51.895	3298.5	16.32	428.41	2763.8
MSF-DiffDet (Ours)	61.83	22.979	592.8	15.13	301.46	975.2

Although MSF-DiffDet and Dual-LateFusion have nearly identical GFLOPs (22.98 vs. 23.42), MSF-DiffDet exhibits a higher CPU inference latency (301 ms vs. 127 ms). This discrepancy arises because GFLOPs only quantify arithmetic operations and do not capture memory-bound overheads, which dominate CPU execution. Specifically, the raw 2D FFT/IFFT transformation in the LFAD module is computationally cheap on its own (measuring only 0.5 ms in isolation). The additional 174 ms latency is primarily caused by the architectural overheads required for spatial-frequency interaction: (i) the concatenation of dual-stream feature maps causes frequent non-contiguous memory access; (ii) the MixBlock attention modules involve tensor reshaping and batched matrix multiplications; and (iii) the frequency branch processes 3-channel input tensors instead of 1-channel. These operations are memory-bandwidth intensive and are not captured by the FLOP count. On GPU, these overheads are largely hidden by massive parallelism.

### Appendix A.3

Figure A1 provides additional qualitative visualizations of forgery-sensitive stroke-region response maps for genuine and forged handwriting samples. All samples are correctly classified by MSF-DiffDet. The visualizations are consistent with the quantitative comparison in Table 7: on average, genuine samples exhibit weak and diffuse responses along stroke regions, whereas forged samples exhibit strong and spatially concentrated responses. We note that genuine samples are not always uniformly low-response: the first genuine sample in Figure A1 shows moderate activation on a subset of stroke segments, reflecting the intrinsic difficulty of some genuine samples near the decision boundary. Such residual variation is also visible in the quantitative standard deviation of the genuine group in Table 7 (±0.0332), and it does not affect the overall separation between genuine and forged responses.

### Appendix A.4

Table A3 reports the full configuration of the three diffusion-based handwriting generators used in this work. All models are used with their official pre-trained checkpoints and without fine-tuning on our data. WordStylist generates word-level images, whereas DiffBrush and TGC-Diff generate text-line images that are subsequently segmented into word-level and character-level units, respectively, as described in Section 4.1. Parameters not explicitly specified in the official release are marked accordingly.

**Table A3:** Configuration of the three diffusion-based handwriting generators used in this work. All models are used with their official pre-trained checkpoints without fine-tuning on our data.

Configuration	WordStylist	DiffBrush	TGC-Diff
Official repository	[29]	[30]	[31]
Generation granularity	Word-level	Text-line	Text-line
Training data	IAM word images	IAM text-line corpus	Chinese text-line dataset
Base VAE	Stable Diffusion v1.5	Stable Diffusion v1.5	Stable Diffusion 3.5
Latent channels	4	4	16
Checkpoint	ema_ckpt.pt	DiffBrush-ckpt.pt	weight.pt
Sampler	DDPM	DDIM	DDIM
Sampling steps	1000	50	50
Eta	N/A	0.0	0.0
Guidance scale	3	Not used	Not used
Generation resolution	64×256	64×1024	64×N, N≤2048
Random seed	Not fixed	1001	1001

### Appendix A.5

Table A4 reports paired statistical significance tests on Test Set A at the per-image level. AUC differences are evaluated using image-level bootstrap confidence intervals (2000 resamples). Fixed-threshold classification differences use the McNemar exact test on paired per-sample outcomes. All tests operate at the per-image level with n≈3000, so their statistical power is governed by the number of test images, ensuring reliable and comparable significance results across all evaluated models.

**Table A4:** Paired statistical significance tests on Test Set A at the per-image level. AUC differences use image-level bootstrap confidence intervals (2000 resamples); accuracy differences use the McNemar exact test. Positive Δ indicates that MSF-DiffDet outperforms the compared model, or that the later ablation variant outperforms the earlier one. Significance codes: *** p<0.001, * p<0.05, n.s. not significant.

Comparison	ΔAUC	95% CI for ΔAUC	ΔAcc (pp)	McNemar *p*	Sig.
MSF-DiffDet vs. baselines					
vs. ResNet-18	+0.0240	[+0.0201,+0.0279]	+3.64	$3.4\times 10^{-15}$	***
vs. ResNet-50-S	+0.0059	[+0.0021,+0.0097]	+0.73	$7.4\times 10^{-2}$	n.s.
vs. Frequency-Only	+0.0134	[+0.0094,+0.0174]	+1.80	$1.8\times 10^{-7}$	***
vs. Dual-LateFusion	+0.0072	[+0.0033,+0.0111]	+0.85	$8.8\times 10^{-2}$	n.s.
vs. F^3^-Net	+0.0147	[+0.0106,+0.0188]	+1.97	$2.1\times 10^{-8}$	***
vs. CNNDetection	+0.0108	[+0.0067,+0.0149]	+1.45	$2.3\times 10^{-5}$	***
vs. UnivFD	+0.0977	[+0.0907,+0.1047]	+14.69	<$5\times 10^{-4}$	***
Adjacent ablation variants					
M2 vs. M1 (+LFAD)	+0.0058	[+0.0019,+0.0097]	+1.87	$9.2\times 10^{-2}$	n.s.
M3 vs. M2 (+LFS)	+0.0157	[+0.0118,+0.0196]	+0.79	$2.3\times 10^{-2}$	*
M4 vs. M3 (+MixBlock)	+0.0141	[+0.0102,+0.0180]	+1.61	$3.7\times 10^{-4}$	***
M5 vs. M4 (+AF-Skip)	+0.0158	[+0.0119,+0.0197]	+0.91	$6.9\times 10^{-2}$	n.s.

### Appendix A.6

We analyze the complementarity of the three reconstructed spectral components $V_{1},V_{2},V_{3}$ produced by LFAD. Table A5 reports the pairwise Pearson correlation coefficients over all Test Set A samples.

To encourage the three learnable masks to specialize to distinct spectral subspaces, we introduce an orthogonality-regularized variant of LFAD that adds a decorrelation loss to the training objective:

(A1) $$L_{\mathrm{orth}}=\frac{1}{B}\sum_{b=1}^{B}\left(\rho_{12}^{(b)2}+\rho_{13}^{(b)2}+\rho_{23}^{(b)2}\right),$$

where $\rho_{ij}^{\left(b\right)}$ is the Pearson correlation between components $V_{i}$ and $V_{j}$ of the *b*-th sample in a mini-batch. This loss is added to the joint objective with a fixed weight of 1.0. Training uses the same data splits, optimizer, and schedule as the main model.

With this regularizer, the pairwise correlations between the three components remain in the moderate range of 0.27–0.36 (Table A5), confirming that LFAD captures complementary spectral information rather than redundant filters. Classification performance is essentially preserved (Acc 93.32±0.27 vs. 93.49±0.35; AUC 96.96±0.20 vs. 96.30±0.44), and the AUC slightly improves, indicating that the decorrelation constraint does not harm the detection task.

To further verify that the learnable decomposition is beneficial relative to a conventional hand-crafted decomposition, we replace the LFAD module with a fixed radial bandpass decomposition while keeping all other network components identical. The fixed-band baseline achieves 91.35% accuracy and 93.96% AUC, whereas the orthogonality-regularized LFAD reaches 93.32% accuracy and 96.96% AUC. Both differences are statistically significant. The accuracy gap of +1.97 percentage points is confirmed by the McNemar exact test with $p=1.27\times 10^{-4}$, and the AUC gap of +0.0300 has a bootstrap 95% confidence interval of [+0.0241,+0.0359]. This confirms that adaptive spectral weighting provides a clear advantage over fixed hand-crafted bands for handwriting forgery detection.

Integrating the orthogonality-regularized LFAD as the default configuration and re-evaluating all robustness and generalization experiments is a natural next step that we identify as future work.

**Table A5:** Quantitative analysis of the orthogonality-regularized LFAD on Test Set A. Top: pairwise Pearson correlation coefficients between the three reconstructed spectral components $V_{1},V_{2},V_{3}$. Lower correlation indicates higher complementarity. Bottom: overall classification performance. All values are mean ± std over three random seeds.

Metric	Value	Std
Component Correlation		
$V_{1}$ vs. $V_{2}$	0.339	0.031
$V_{1}$ vs. $V_{3}$	0.269	0.024
$V_{2}$ vs. $V_{3}$	0.356	0.030
Classification Performance		
Accuracy (%)	93.32	0.27
AUC (%)	96.96	0.20
EER (%)	10.78	0.25

### Appendix A.7

Table A6 reports calibration, threshold, and uncertainty statistics for MSF-DiffDet across Test Sets A, B, and C, aggregated over three training seeds. ECE is computed with 10 equal-width bins. FPR and FNR are obtained by applying the fixed validation-set EER threshold $\tau_{val}$ to each test set. Note that at this operating point FPR and FNR are generally not equal, so they cannot be directly averaged to estimate the threshold-independent EER reported in Table 3 and Table 10. Confidence denotes the mean maximum softmax probability, reported separately for correctly and incorrectly classified samples.

**Table A6:** Calibration, threshold, and uncertainty statistics for MSF-DiffDet across Test Sets A, B, and C, aggregated over three training seeds. ECE: Expected Calibration Error (10 bins, lower is better). FPR and FNR are computed at the fixed validation-set EER threshold $\tau_{val}$. Because $\tau_{val}$ is not the test-set EER operating point, FPR≠FNR at this threshold, and their average does not equal the threshold-independent EER reported in Table 3 and Table 10. Confidence denotes the mean maximum softmax probability.

Test Set	ECE (%)	FPR@$\tau_{\mathrm{val}}$ (%)	FNR@$\tau_{\mathrm{val}}$ (%)	Conf. Correct	Conf. Incorrect
Test Set A	0.38±0.11	6.22±0.38	6.80±0.42	0.9741±0.0012	0.7624±0.0261
Test Set B	4.00±0.65	13.85±1.05	7.83±0.86	0.9809±0.0035	0.8584±0.0103
Test Set C	25.12±1.15	29.72±2.20	40.52±2.35	0.9415±0.0210	0.8827±0.0275

### Appendix A.8

To isolate generator shift from script and dataset shift, we train on DiffBrush-generated handwriting and test on WordStylist-generated handwriting, using the LOGO split described in Section 4.1.2. All four models are trained under identical settings (AdamW, cosine annealing, three random seeds 2024/2025/2026). We select four representative models covering the spatial-only, frequency-only, dual-stream, and foundation-model families; the remaining baselines in Table 3 are not re-trained in the LOGO setting because they fall into the same families and would not change the comparison. AUC and EER are reported as the primary metrics because the fixed 0.5 threshold calibrated on the source generator is suboptimal on the unseen target generator. The results are summarized in Table A7.

**Table A7:** Leave-one-generator-out (LOGO) cross-generator evaluation. Train: DiffBrush. Test: WordStylist. Writer-disjoint and generator-disjoint. Results are mean ± std (%) over three random seeds.

Model	Acc	Precision	Recall	F1	AUC	EER↓
MSF-DiffDet (Ours)	54.58 ± 0.33	99.02 ± 0.08	9.41 ± 0.74	17.12 ± 1.23	84.74 ± 0.98	21.69 ± 1.10
ResNet-50 (Spatial-Only)	52.84 ± 0.31	99.41 ± 0.06	5.81 ± 0.62	10.92 ± 1.01	81.68 ± 1.22	23.10 ± 0.95
Frequency-Only	53.01 ± 0.59	99.35 ± 0.27	6.05 ± 1.17	11.38 ± 2.10	77.90 ± 1.81	28.60 ± 1.96
UnivFD	51.22 ± 0.04	62.86 ± 0.36	5.98 ± 0.06	10.92 ± 0.10	56.24 ± 0.13	45.54 ± 0.06

MSF-DiffDet achieves the best cross-generator AUC (84.74%) and the lowest EER (21.69%), followed by ResNet-50 (Spatial-Only, 81.68%), Frequency-Only (77.90%), and UnivFD (56.24%). The ranking differs from the in-generator setting: the purely spatial ResNet-50 outperforms the purely frequency-domain variant, indicating that spatial texture features provide the primary cross-generator transfer signal while frequency-domain features partially overfit to generator-specific spectral artifacts. The dual-stream MSF-DiffDet yields the best cross-generator performance. The low fixed-threshold recall (0.06–0.09) across all models reflects a probability-distribution shift rather than a failure of ranking capability.

### Appendix A.9

To examine whether the detector relies on interpolation-specific artifacts, we evaluate the trained MSF-DiffDet checkpoints on Test Set A under four resize interpolation modes (bilinear, bicubic, nearest, Lanczos), with all images resized to a fixed 224×224 resolution. The results are reported in Table A8.

**Table A8:** Interpolation control experiment. All results are mean ± std (%) over three random seeds.

Interpolation	AUC (%)	EER (%)↓	BA (%)
Bicubic	96.98 ± 0.02	10.51 ± 0.42	86.78 ± 4.82
Bilinear	96.81 ± 0.08	12.09 ± 0.47	85.51 ± 4.70
Lanczos	96.98 ± 0.02	10.40 ± 0.40	87.21 ± 4.44
Nearest	96.94 ± 0.07	11.07 ± 0.68	87.25 ± 3.09

The AUC varies within a narrow range of 0.17 percentage points across the four interpolation modes (96.81–96.98%), with the fluctuation amplitude remaining small relative to the inter-seed standard deviation. This indicates that the detector’s discriminative power is not primarily driven by interpolation-specific artifacts. Because the fixed 224×224 resolution is applied identically to genuine and forged samples, any residual resolution-related cue would be shared by both classes and could not alone explain the classification performance. The larger standard deviation of the balanced accuracy (3–5 percentage points) compared with AUC (0.02–0.08 percentage points) reflects the sensitivity of fixed-threshold metrics to probability-distribution shifts across seeds, and further motivates the use of threshold-independent AUC and EER as the primary metrics.

We note that EER is computed from the full ROC curve via min|FNR−FPR| and is not constrained to equal 1−AUC under sparse ROC sampling. The model outputs are highly concentrated near 0 and 1, so the ROC curve is sparse in the intermediate region, which accounts for the difference between EER and 1−AUC. This is a numerical property of the ROC sampling rather than a calculation error.

To visually inspect the learned frequency decomposition, Figure A2 displays the three learnable Fourier masks $M_{1},M_{2},M_{3}$. Because the raw masks are initialized as all-ones and the absolute values are dominated by the central low-frequency component, we plot their weight deviations from the uniform initialization (i.e., weight minus 1.0). The red regions indicate frequencies where the mask amplifies the input spectrum, and the blue regions indicate suppression. As shown, the network does not hard-code a strict low/mid/high partition; rather, it learns a set of complementary, orthogonal frequency subspaces that focus on the mid-to-low frequency bands. This adaptive spectral selection is precisely what allows the model to capture the subtle stroke boundary irregularities of diffusion-generated handwriting.

### Appendix A.10

To isolate the effect of the localization supervision, we train a variant of MSF-DiffDet where the localization loss weight is set to λ=0. The network architecture and all other hyper-parameters are kept unchanged. As reported in Table A9, removing the localization supervision reduces the in-domain Test Set A AUC from 96.30% to 95.60% (−0.70 pp). In the strict LOGO cross-generator setting, however, the AUC plummets from 84.74% to 70.99% (−13.75 pp), and EER increases drastically from 21.69% to 35.04%. The cross-generator drop is approximately 20 times larger than the in-domain drop, confirming that the spatial supervision acts as a crucial regularizer that helps the shared encoder learn generator-agnostic, region-level forensic cues.

**Table A9:** Ablation on the localization loss. “$\lambda_{\mathrm{loc}}=0$” removes the localization supervision while keeping the network architecture unchanged. Results are mean ± std (%) over three random seeds.

Setting	Model	AUC (%)	EER (%)↓
Test A	MSF-DiffDet ($\lambda_{\mathrm{loc}}=5$)	96.30 ± 0.44	10.45 ± 0.22
	MSF-DiffDet ($\lambda_{\mathrm{loc}}=0$)	95.60 ± 0.51	11.55 ± 0.39
LOGO	MSF-DiffDet ($\lambda_{\mathrm{loc}}=5$)	84.74 ± 0.98	21.69 ± 1.10
	MSF-DiffDet ($\lambda_{\mathrm{loc}}=0$)	70.99 ± 1.43	35.04 ± 1.87

Furthermore, to verify that the response map encodes sample-specific evidence rather than a label-conditioned global pattern, Figure A3 visualizes the response maps of four correctly classified forged samples sharing the same image label. Although all four samples are forged and classified with high confidence, their high-response regions (yellow) are distributed non-uniformly along the strokes, localizing specific boundary irregularities and high-curvature segments while leaving other parts at moderate response. This confirms that the response map does not merely perform stroke segmentation; it learns to localize sample-specific forensic evidence.
